# Methylglyoxal in Cardiometabolic Disorders: Routes Leading to Pathology Counterbalanced by Treatment Strategies

**DOI:** 10.3390/molecules28237742

**Published:** 2023-11-24

**Authors:** Izabela Berdowska, Małgorzata Matusiewicz, Izabela Fecka

**Affiliations:** 1 Department of Medical Biochemistry, Wroclaw Medical University, 50-368 Wroclaw, Poland; malgorzata.matusiewicz@umw.edu.pl; 2 Department of Pharmacognosy and Herbal Medicines, Wroclaw Medical University, 50-556 Wroclaw, Poland

**Keywords:** methylglyoxal, glyoxalase, advanced glycation end products, MG-H1, metabolic syndrome, insulin resistance, diabetes mellitus, cardiovascular disease, metformin, methylglyoxal scavengers

## Abstract

Methylglyoxal (MGO) is the major compound belonging to reactive carbonyl species (RCS) responsible for the generation of advanced glycation end products (AGEs). Its upregulation, followed by deleterious effects at the cellular and systemic levels, is associated with metabolic disturbances (hyperglycemia/hyperinsulinemia/insulin resistance/hyperlipidemia/inflammatory processes/carbonyl stress/oxidative stress/hypoxia). Therefore, it is implicated in a variety of disorders, including metabolic syndrome, diabetes mellitus, and cardiovascular diseases. In this review, an interplay between pathways leading to MGO generation and scavenging is addressed in regard to this system’s impairment in pathology. The issues associated with mechanistic MGO involvement in pathological processes, as well as the discussion on its possible causative role in cardiometabolic diseases, are enclosed. Finally, the main strategies aimed at MGO and its AGEs downregulation with respect to cardiometabolic disorders treatment are addressed. Potential glycation inhibitors and MGO scavengers are discussed, as well as the mechanisms of their action.

## 1. Methylglyoxal in (Patho)physiology

Methylglyoxal (MGO) is the major compound belonging to α-dicarbonyl molecules, which are termed “reactive carbonyl species” (RCS) responsible for “carbonyl stress”. They are highly reactive compounds that easily modify biological macromolecules, including peptides, proteins, lipoproteins, and nucleic acids via the generation of advanced glycation end products (AGEs) [1]. Therefore, together with other irritable molecules like reactive oxygen and nitrogen species (RONS), they disturb the functioning of cellular organelle, thus stimulating their rearrangements, leading to autophagy, apoptosis, or proliferation of cells. Such phenomena, when not counteracted by detoxifying mechanisms, stimulate oxidative stress and enhance inflammatory processes, contributing to the development of a variety of pathological conditions [1,2,3].

However, because MGO is constantly produced in the organism as a glycolytic byproduct, it might be also involved in beneficial processes stimulating and maintaining protective mechanisms to prepare the organism for handling with enhanced/pathological concentrations of RCS and RONS. Such a phenomenon, known as hormesis, is observed when low vs. high doses of a factor yield opposite effects, e.g., a high concentration of a compound is harmful, whereas at a low concentration, it works in a beneficial manner [4]. Recent data point to MGO playing such a dual function in organisms [1]. Although it is toxic at high levels [5], when MGO is generated or tested at low concentrations, it seems to stimulate protective mechanisms, including the upregulation of heat shock proteins involved in handling misfolded proteins [6] or the activation of the proteasomal system participating in the removal of damaged proteins (yielding the extension of the healthy lifespan of *C. elegans*) [7].

A normal MGO level in human blood plasma has been estimated at 0.06–0.25 µM, whereas its cellular concentration reaches 1–5 µM [5]. In metabolic disorders mostly associated with hyperglycemia (such as metabolic syndrome and diabetes), MGO, as well as its main end product (D-lactate) and MGO-derived AGEs (MAGEs), usually undergo upregulation intertwined in pathological processes [8,9,10,11,12,13,14,15,16,17,18,19,20,21,22,23,24].

### 1.1. Endogenous Sources of MGO

MGO is endogenously produced under physiological conditions, in which its main source (around 90%) are trioses derived from glycolysis: dihydroxyacetone phosphate and glyceraldehyde-3-phosphate [5,25,26,27]. They undergo a nonenzymatic conversion into MGO via an intermediate enediolate phosphate [26]. Around 0.09–0.4% of glycolytic flux is probably associated with MGO generation [28,29]. This pathway seems to be stimulated under hyperglycemic conditions due to the fact that the major MGO precursors are glucose (Glc) and fructose (Fru) [30,31,32]. In their recent report, Zhang et al. [32] have shown that both blood plasma and tissue MGO levels rose in parallel to Glc during an oral glucose tolerance test. Additionally, the authors observed the increase in MGO-modified proteins in the circulation, which confirms Glc to be the main source of MGO.

Fructose has drawn attention with respect to its deleterious effects implicated in metabolic syndrome development, including obesity, insulin resistance, and hypertension [33,34,35]. In comparison with glucose, fructose is not so tightly regulated by hormones (e.g., insulin). When it enters the glycolytic pathway in the liver (the organ responsible for around 90% of its metabolism), it overcomes regulatory steps limiting glucose degradation (glucokinase and phoshofructokinase), which easily yields trioses accumulation (being converted into diacylglycerol (DAG), triacylglycerol (TAG), and methylglyoxal (MGO)) [34]. Fructose excess in the liver leads to unfavorable processes, such as uric acid generation, lipogenesis, and gluconeogenesis, hence stimulating proinflammatory pathways. On the other hand, an excess of glucose is utilized for glycogen generation—the main carbohydrate energy storage in the liver and muscles. Additionally, fructose is more vulnerable to nonenzymatic oxidation, 8–10 times more active in the formation of AGEs than Glc, and (although present at levels around 100 times lower in the blood plasma) I under some pathological conditions, it may be the main source of MGO [34]. Except for its detrimental effects on the liver, fructose also disturbs the functions of the adipose tissue, inducing leptin-resistance, adipogenesis, oxidative stress, and inflammation. Such fructose-triggered deleterious pathways are highly probably the consequences of an overload of fructose in the diet, especially in the form of a high-fructose syrup, being a commonly applied additive in many highly processed foodstuffs, such as nonalcoholic beverages. Accordingly, the fructose-rich diet has been connected with metabolic disturbances leading to obesity, dyslipidemia, metabolic syndrome, and type 2 diabetes (promoting insulin resistance and gluconeogenesis), as well as nonalcoholic fatty liver disease (NAFLD) and cardiovascular disease (CVD) [33,34,36]. One of the factors linking fructose overload and many of the above-mentioned pathological processes may be the excessive production of MGO. As hypothesized by Gugliucci [37], an excess of dietary Fru (whose increasing intake is paralleled with metabolic syndrome prevalence) would lead to the accumulation of MGO in the liver, which, in turn, would modify 3 Arg residues in AMP-activated kinases (AMPKs). Because AMPKs are the energy sensors of the cells, they are activated at a low energy level (reflected by AMP increase) and stimulate catabolic pathways, leading to energy replenishment. Fru influx into the liver and its entering glycolysis leads to the depletion of ATP (used for its phosphorylation) associated with an increase in AMP. This should stimulate catabolic pathways and inhibit anabolic pathways (via AMPKs activation by AMP). However, experimental data indicate quite the opposite regulation-accelerating processes of synthesis (lipogenesis, gluconeogenesis) in Fru overload conditions. Hence, Gugliucci has put forward the hypothesis that it might be MGO-modified AMPK that loses its function (because MGO modification makes it insensitive to AMP regulation), otherwise leading to the acceleration of opposite processes. Finally, instead of the degradation/oxidation of macromolecules to gain energy, their synthesis is enhanced, yielding hyperglycemia and/or liver steatosis, with further consequences [37].

The minor endogenous sources of MGO include amino acids, glycerol, and ketone bodies, as well as glycated proteins [5,26,38,39,40]. For example, MGO may be generated from aminoacetone (derived from threonine or glycine catabolism), deamination [41], or the degradation of glucose-glycated proteins [38]. Additionally, lipid peroxidation products (aldehydes and ketoaldehydes) give rise to the production of MGO [39]. Therefore, under pathological conditions stimulated by a fructose-rich diet and associated with oxidative stress, hyperglycemia, and an overproduction of ketone bodies (observed in disturbances connected with metabolic syndrome, diabetes, and cardiovascular complications), multiple routes of MGO generation are possible [40].

### 1.2. Exogenous Sources of MGO

MGO, other α-dicarbonyl compounds, and their AGEs have been detected in dietary products, especially highly processed foodstuffs that have been subjected to high temperatures, such as dairy products [39,42,43]. For example, MGO can be found in cookies, alcoholic beverages, soy sauce, coffee, and honey [43,44,45,46,47]. However, exogenous MGO sources do not seem to contribute significantly to the total MGO load in the human body due to its putative degradation in the gastrointestinal tract (GI) and detoxification by the glyoxalase system in the epithelial cells lining GI lumen [48]. Nevertheless, deleterious MGO effects can be observed in the GI tract, both via the impact of MGO-glycated foods on the composition of intestinal microbiome, as well as the metabolism of dietary carbohydrates by bacteria, which can lead to MGO formation [39].

### 1.3. MGO Modification of Macromolecules

#### 1.3.1. MGO-Derived AGEs (MAGEs)

MGO is the major α-dicarbonyl compound involved in the modification of peptides, proteins, and lipoproteins, resulting in AGEs formation (MAGEs). It modifies arginine (Arg), lysine (Lys), and cysteine (Cys) residues in macromolecules, showing the greatest efficiency for Arg alterations [49]. MGO irreversibly reacts with the Arg guanidine group, generating several types of derivatives, including three cyclic hydroimidazolones: MG-H1, MG-H2, and MG-H3 [5,50,51]. The most prevalent is MG-H1 isoform, which is responsible for more than 90% of MGO alterations [52]. Both MG-H1 and MG-H2 have been detected in human lens proteins [53], but when antibodies against hydroimidazolones have been tested on human endothelial cells (Ea.hy 926 cells), only MG-H1 and MG-H3 were identified (and their nuclear localization was reported in that study) [54]. Similarly, only MG-H1 and a derivative of MG-H3 (CEA) were detected in the chromatin from human epithelial cells (HEK293), and their presence was observed in the chromatin from several other human cell lines, as well as murine tissues from many organs [55]. Except for hydroimidazolones, other derivatives of MGO-modified Arg include tetrahydropyrimidine (THP) and argpyrimidine (AP) [56,57]. Besides Arg, MGO is able to modify the Lys side chain, yielding its carboxyethyl derivative (CEL) or forming Lys dimers (MOLD), as well as cross-linking Arg with Lys to generate MODIC adducts [5].

**Figure 1:**
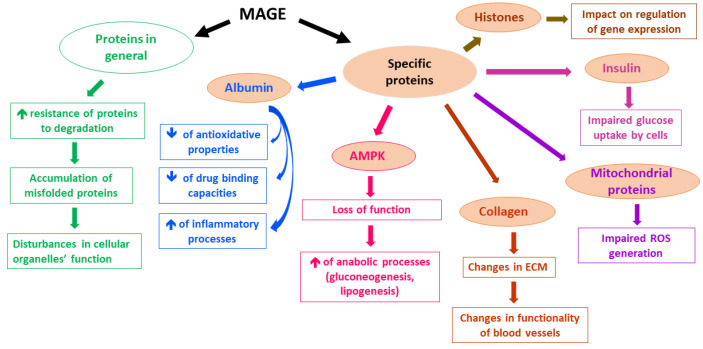
Examples of MAGEs effects on the selected molecular and cellular processes.

A lot of proteins have been reported to undergo MGO-derived Arg modifications, which leads to their impaired functioning. Although in many experimental studies applied concentrations of MGO far exceeded physiological levels of MGO, low MGO levels also seem to alter the functionality of proteins [5]. For example, MGO-modified Arg_410_ (yielding MG-H1 residues) in albumin [58] probably disturbs a drug-binding function, as well as the esterase activity of this protein [59]. Additionally, MGO-glycated albumin shows decreased antioxidative potential [60] and seems to stimulate inflammatory processes via the mobilization of such cytokines as TNF-α [61,62] and IL-1β [63]. Other proteins whose functions can be disturbed upon MGO glycation include collagen [64,65], hemoglobin [66,67,68], insulin [69], and mitochondrial proteins, whose impairment leads to ROS generation [70]. Moreover, MAGEs formation interferes with proteolysis coupled with lysosomal and proteasomal systems [5]. On the one hand, an extensive protein glycation makes proteins resistant to degradation, whereas on the other hand, MGO impact on the ubiquitination process might enhance the proteasomal degradation of some proteins (less probable in vivo, however, due to greater than physiological MGO levels tested in the experiments) [5]. Therefore, MGO-glycation, especially under hyperglycemic conditions, seems rather to impair the functioning of proteolytic systems, leading to the accumulation of misfolded proteins in the cells, followed by disturbances in intracellular organelles [5]. Such a phenomenon has been observed in Glo1 knockdown mice, in which the MGO glycation of a proteasomal subunit decreased proteolytic activity [71]. Furthermore, MAGEs modification of histones seems to affect the epigenetic regulation of gene expression. Histones’ side chains of Arg and Lys altered by MGO (yielding MG-H1, MG-H3/CEA, and CEL derivatives) led to the increase or decrease in transcription of multiple genes [55]. Hence, the potential effect of MAGEs on genes expression might lead, via multiple pathways, to pathology, enhancing deleterious processes, especially in metabolic syndrome associated with hyperglycemia and diabetes, in which it might contribute to the development of hyperglycemic legacy effect (metabolic memory) [72]. The examples of MAGEs effects on selected proteins and their consequences are presented in Figure 1.

#### 1.3.2. MGO-Derived DNA Modifications

In comparison with protein glycations by MGO, much less is known about nucleic acid modifications [5]. The most reactive nucleoside is deoxyguanosine, which, upon MGO action, yields CEdG and MG-dG derivatives [5,27]. CEdG is more abundant and stable, so it seems to play a more important role with reference to MGO-associated pathologies [5], mainly metabolic syndrome and diabetes, in which CEdG increase has been observed in animal models [12,13] and diabetic patients’ tissues [73], as discussed in the following chapters.

### 1.4. MGO Scavenging System

MGO undergoes detoxification reactions catalyzed by a ubiquitous glyoxalase system composed of glyoxalase 1 (Glo1) and 2 (Glo2), yielding D-lactate [14]. The first enzyme Glo1 requires reduced glutathione (GSH) for the production of an intermediate (lactoylglutathione), whereas Glo2 catalyzes lactoylglutathione conversion into D-lactate, which is coupled with the regeneration of GSH [27]. Abundant in cytosol, Glo1 is characterized by a high specificity toward MGO and catalyzes the rate-limiting reaction in MGO metabolism [27]. Glo2, except for being located in the cytosol, is also present in the mitochondrion [26].

Interestingly, DJ-1 (PARK7 = Parkinson’s disease protein 7) has been also suggested to be involved in MGO detoxification [74]. Whereas a mutated *DJ-1* gene is implicated in up to 1% of early onset Parkinson’s disease cases [75], its normal product is a multifunctional protein that controls the activity of mitochondria (being engaged in mitophagy) [75]. It is also a sensor of the cellular oxidative stress, upon which it is activated and, in turn, switches on protective mechanisms, e.g., controlling the expression of antioxidative enzymes [76]. Additionally, DJ-1 may play a role in MGO degradation due to its glyoxalase activity (less certain because this activity is low in comparison with Glo1), as well as in the repairment of MGO-glycated proteins and nucleic acids, because it may also show deglycase activity [55,77,78] (a more probable function) [5]. However, Pfaff et al. [79], in their *DJ-1* knockdown and knockout fruit fly models, have questioned the function of this protein in MGO detoxification.

Overall, this is the glyoxalase system (Glo1 and Glo2) that contributes mainly to MGO scavenging (metabolizing more than 98% of MGO) [5]. Additionally, MGO may also enter other pathways of degradation, yielding pyruvate (when catalyzed by NADPH-dependent aldehyde dehydrogenases (ALDHs)) or hydroxyacetone (when catalyzed by aldoketo reductases (AKRs)) [5]. The importance of AKRs in MGO scavenging, which are associated with protection from AGEs formation and atherosclerotic lesion generation, has been reported by Baba et al. [80]. Therefore, these minor routes of MGO detoxification may play a role in pathological processes partially taking over the functions of glyoxalases whose downregulation is observed under cellular stress [27]. Such a compensatory mechanism has been shown by Schumacher et al. [81] and Morgenstern et al. [82] in Glo1 knockout experimental models.

## 2. MGO and MAGEs in Metabolic Syndrome and Diabetes

### 2.1. Metabolic Syndrome

Metabolic syndrome is a set of disturbances associated with defects in lipid and carbohydrate metabolism. This syndrome is diagnosed in individuals who present any three out of five characteristics, namely, an enhanced concentration of triacylglycerols, elevated glucose, a decreased level of HDL-cholesterol, hypertension, or adiposity connected with an excessive level of visceral/liver fat. A characteristic feature of individuals suffering from metabolic syndrome is insulin resistance and chronic low-grade inflammation [83], which can develop further into disorders such as type 2 diabetes mellitus (T2DM) and cardiovascular conditions [84], including coronary heart disease and stroke [85].

### 2.2. MGO and MAGEs in Metabolic Syndrome and Diabetes in Animal Models and Cell Cultures

Experimental models used to estimate the association of MGO and MAGEs with metabolic disturbances include MGO- or fructose-fed animals, genetically modified animals that develop obesity, diabetic and atherosclerotic characteristics, as well as glyoxalase 1-deficient or -overexpressing animals. Additionally, spontaneously hypertensive rats (SHR) rats have been used to examine the pathological background underlying hypertension. One of such models used in metabolic syndrome/diabetes studies is genetically modified mice that highly express defective gene coding for the leptin receptor (*Lepr*^db/db^); hence, they develop leptin resistance leading to obesity, hyperinsulinemia, and hyperglycemia [86,87]. In search of a diagnostic marker that could be used in the diagnosis of prolonged diabetes, Jaramillo et al. [12] found that MGO-modified deoxyguanosine (CEdG) was significantly elevated in the urine and tissues of hyperglycemic *Lepr*^db/db^ mice in comparison with normoglycemic animals. A similar increase in urinary CEdG has been observed in diabetic (T1DM) rats [13]. Additionally, urinary CEdG has been shown to be an independent prognostic factor of hyperglycemia and was positively correlated with fasting plasma glucose (FPG) in hyperglycemic animals and with HbA1c in all animals [12]. Furthermore, two protein (M)AGEs-lysine derivatives (CML and CEL) were elevated in the urine of hyperglycemic mice, but they did not correlate with FPG. Nevertheless, a positive correlation was reported between CEdG and both CML and CEL in hyperglycemic mice [12]. Hence, the authors suggested CEdG to be a promising marker in metabolic diseases.

Elevated levels of both MGO and D-lactate (the end product of MGO metabolism by the glyoxalases system) have been reported in T1DM rats in the lens and blood [14]. Additionally, MGO concentration was increased in those animals’ kidneys. Furthermore, MGO treatment has impaired the glycemia and lipid profile both in lactating rat mothers (in their blood plasma and breast milk) and their adult male offspring who showed features of obesity [88]. Therefore, MGO seems to be implicated both in type 1 and 2 diabetes.

To elucidate which metabolic pathways are responsible for the overgeneration of MGO and its deleterious effects in pathology, Liu et al. [89] have examined four different rat models with metabolic syndrome features, complemented with experiments on vascular smooth muscle cells (VSMCs). In both the rats’ aortas and their VSMCs, the authors reported the upregulation of enzymes responsible for fructose degradation, as well as fructose-specific transporter (Table 1). These effects were stimulated using a high Fru level, augmented by insulin, and led to an increase in MGO. Additionally, a high Glc level seemed to contribute to MGO generation via Fru production (polyol pathway) rather than glycolysis [89]. Thus, the authors underlined the causative importance of Fru associated with MGO generation and the further deleterious consequences in obesity, hypertension, and diabetes with cardiovascular impact, especially in light of the Fru-rich diet common in well-developed countries. The same type of VSMC has been shown to develop oxidative stress upon MGO exposure [90,91]. MGO increased the level of RONS through its deleterious effect on the respiratory chain (impairing complex III activity, which was associated with superoxide anion generation and decrease in ATP synthesis), as well as the inhibition of superoxide scavenging enzyme manganese superoxide dismutase (MnSOD) [91]. As discussed in the following chapters, RONS overgeneration is implicated in pathologic routes leading to cardiometabolic disorders. Similarly, a chronic low-grade inflammatory state is associated with metabolic syndrome, diabetes, and CVD, and MGO has been shown to mediate macrophages-induced proinflammatory processes, leading to the development/deepening of inflammation [92,93].

#### MGO/MAGEs in Insulin Resistance Development

Insulin resistance, a condition observed in metabolic syndrome and T2DM and implicated in cardiometabolic disorders, is characterized by the impairment of insulin-triggered signaling pathways, which leads to disturbances in the insulin-controlled metabolism of carbohydrates and lipids, as well as endothelial dysfunction. The main organs affected by insulin resistance include the liver, adipose tissue, skeletal muscles, endothelium, and pancreas.

As discussed by Nigro et al. [112] and Shamsaldeen et al. [113], MGO accumulation in pathology is implicated in insulin resistance development both through the modification of this hormone molecule itself and the components of its signaling pathways.

In skeletal muscles, insulin resistance is mainly characterized by a decreased Glc uptake caused by the inefficient mobilization of Glc transporters (GLUT-4), which are normally increased upon insulin induction. MGO has been shown to accumulate in metabolically impaired skeletal muscles as a result of the lowered efficiency of its main scavenging system (glyoxalases) [114]. An excess of MGO disturbs insulin signaling and promotes oxidative and inflammatory processes, which is associated with mitochondrial damage (including mitochondrial DNA), MAGE formation (MG-H1), and structural changes in muscle proteins [114].

In search of the effect of MGO on insulin resistance in skeletal muscles, MGO-exposed and insulin-stimulated rat myoblasts have been examined [115,116]. Whereas a short-term exposure to a high concentration of MGO decreased Glc uptake by the cells (probably through the modification of IRS-1, which lowered its insulin-induced tyrosine phosphorylation, followed by the impairment of PI3K mobilization and PKB phosphorylation) [115], longer exposure to low MGO levels caused increased Glc uptake [116]. In the latter study, MGO was shown to interfere with GLUT-4 transporter translocation, diminishing their endocytosis and hence, increasing their number on the myocytes’ surfaces. Although MGO-induced ROS generation was observed in these cells, MGO’s effect on GLUT-4 seemed to be independent of oxidative stress. Additionally, the MGO-induced apoptosis of myocytes, as well as GLUT-4 modification (with MG-H1 formation), was reported in this study [116]. These observations indicate MGO interference with Glc uptake by skeletal muscle cells. However, neither an impact on insulin receptor autophosphorylation, serine/threonine phosphorylation of IRS-1, nor Akt phosphorylation were found upon MGO treatment [115,116].

Visceral adiposity associated with metabolic syndrome and further complications leads to the initiation of chronic inflammation (connected with a shift toward proinflammatory macrophages yielding the secretion of IL-6 and TNF-α), disturbances in the adipokines profile (augmented leptin secretion paralleled by decreased adiponectin), and insulin resistance development [83]. The impact of MGO on these processes has been studied in rodent adipocytes, in which its inhibitory effects on Glc uptake, IRS-1 tyrosine phosphorylation, and PI3K kinase activity were observed [94,100,103]. Additionally, MGO-fed rats developed some pathological feature characteristic for insulin resistance and diabetes, such as lowered insulin sensitivity, enhanced free fatty acids levels, and decreased adiponectin in the circulation, as well as proapoptotic, profibrotic and proinflammatory characteristics in the adipose tissue [99,106,107,117] (Table 1). However, not all of the data coming from experiments on MGO-fed rats reported the impairment of glycemia or insulinemia [97] (Table 1). Other MGO-induced disturbances in the adipose tissue included the impairment of blood vessel formation associated with increased hypoxia [117]. Additionally, MGO seemed to stimulate (adrenaline-induced) lipolysis [107], which might be mediated by the MGO-caused degradation of perilipin A [97,117] (Table 1). Because perilipins are proteins stabilizing lipid droplets and protecting them from lipases [118], their decrease would lead to the enhanced hydrolysis of triacylglycerols, yielding an efflux of free fatty acids into circulation.

Similarly, MGO-injected mice have developed systemic insulin resistance resulting from the impairment of the insulin-triggered signaling pathway, as observed in the murine aortas and endothelial cells [101] (Table 1). MGO treatment caused the suppression of insulin-induced pathway leading through the activation of IRS-1, Akt, and eNOS, probably partially via the induction of ERK ½, which inhibited IRS-1. In this way, MGO seems to disturb the balance of the processes yielding vasorelaxation (NO production) and the routes ending up with vasoconstriction (endothelin-1 production) in favor of the latter [101].

MicroRNA oligonucleotides (miRNAs) are responsible for the posttranscriptional regulation of the components of multiple signaling pathways, including insulin-triggered routes; hence, they are implicated in different disorders encompassing cardiometabolic diseases [119,120]. In search of the elucidation of miRNAs’ role in MGO-induced insulin resistance in endothelial cells, Mirra et al. [121] performed diabetes-associated miRNA profiling in murine endothelial cells exposed to MGO. They found four downregulated miRNAs, of which two (miR-190a and miR-214) affected MGO-induced insulin resistance in endothelium, leading to its dysfunction [121,122]. MGO seems to impair insulin-triggered pathway (IRS1/Akt/eNOS/NO release) through the downregulation of miR-190a, which in turn, is associated with KRAS GTPase upregulation. The inhibitory effect of MGO on miR-190a may be connected with its modification/activation of histone deacetylase (HDAC), thus epigenetically restraining miR-190a synthesis [121]. Similarly, MGO-caused miR-214 downregulation is associated with a disturbance in insulin signaling, as shown by its effect on Akt activity [122]. Namely, miR-214 seems to be a negative regulator of PH domain leucine-rich repeat protein phosphatase 2 (PHLPP2), the enzyme that inactivates Akt via its dephosphorylation. miR-214 inhibition (comparable with MGO exposure) led to a 4-fold increase in PHLPP2, which attenuated the insulin-induced Akt pathway in murine endothelial cells [122]. Therefore, it might be suggested that MGO’s effect on endothelial cells is mediated by the downregulation of two miRNAs (miR-190a and 214), followed by the inhibition of the insulin-triggered Akt pathway, shifting the balance from vasodilation toward vasoconstriction due to decreased NO generation.

Except for disturbing the downstream components of the insulin signaling pathway, MGO seems to modify the insulin molecule itself, as reported by Jia et al. [69]. In light of these authors’ findings, it is MGO-modified insulin that impairs Glc uptake both by adipocytes and skeletal muscle cells, rather than free MGO. Additionally, MGO-modified insulin lost the ability to attenuate insulin release using pancreatic β-cells (impaired feedback inhibition) and was inefficiently cleared by hepatocytes [69].

The impact of MGO on insulin- or Glc-stimulated signaling and its consequences in rat pancreatic β-cells was investigated by Fiory et al. [123]. The authors observed that MGO inhibited insulin secretion by Glc-induced pancreatic β-cells, which was associated with the attenuation of PKB activation. Additionally, the MGO-caused inhibition of several components of the insulin-triggered signaling pathway was found (IRS, PI3K, PKB, GSK-3), as well as the reverse by the MGO insulin- and Glc-induced upregulation of three genes coding for pancreatic duodenal homeobox-1, insulin, and glucokinase. The impairment of the insulin signaling pathway was probably associated with the MGO modification of IRS because CEL and AP adducts were detected on this protein upon MGO exposure [123]. A similar inhibitory effect of MGO on insulin secretion by pancreatic β-cells upon Glc induction was observed by Bo et al. [124]. However, different signaling routes were analyzed in the latter study, namely, those leading through ROS generation and MAPK pathway upregulation. MGO-induced oxidative stress and apoptosis and these effects were associated with the upregulation of uncoupling protein 2 (UCP-2), a decrease in mitochondrial membrane potential and ATP synthesis, and an increased expression and activation of JNK and P-38 kinases, finally resulting in insulin secretion inhibition [124].

Therefore, MGO seems to be involved in insulin resistance development through the modification of the hormone molecule in circulation and the alteration of insulin-triggered signaling components intracellularly (in endothelial cells, adipocytes, myocytes, and pancreatic β-cells), which impairs pancreas functionality, as well as insulin-regulated vascular homeostasis and the metabolism of lipids and carbohydrates in the adipose tissue and skeletal muscles. MGO-affected components probably include IRS-1, PI3K, and PKB/Akt, but not the insulin receptor [69,94,100,103,115,123]. Moreover, the MAPK pathway, oxidative stress, and UCP-2 upregulation coupled with mitochondrial dysfunction and apoptosis triggering—all caused by MGO—probably lead to pancreatic β-cell impairment [124].

Due to the inhibitory effects on Glc and the removal of insulin from circulation, MGO/MAGE seem to be important factors contributing to hyperglycemia and hyperinsulinemia. On the other hand, MGO might also contribute to the reduction of insulin in circulation, a phenomenon observed in later stages of T2DM. This effect has been reported by Dhar et al. [103], who found out that MGO treatment in rats diminished Glc uptake by pancreatic cells, enhanced their apoptosis, and inhibited insulin secretion (Table 1).

### 2.3. MGO, Its Metabolic Products, and MAGEs in Patients with Metabolic Syndrome and Diabetes

Studies on MGO’s impact on the pathomechanisms of disease development mostly focus on its participation in the development and perpetuation of metabolic syndrome and diabetes with their macro- and micro-complications.

In vitro studies on red blood cell suspension indicate that the culture levels of MGO, S-D-lactoylglutathione, and their end-metabolite, D-lactate, were elevated under hyperglycemic conditions [28]. At the same time, the activities of Glo1 and Glo2 did not exhibit any elevation. This led to the conclusion that periodic hyperglycemia may lead to the development of complications associated with diabetes. Indeed, subsequent studies revealed that the systemic concentrations of MGO, S-D-lactoylglutathione, and D-lactate are elevated in diabetic patients, as compared to healthy subjects, pointing to the increased flux of metabolites through the glyoxalase system [10,16,18,19]. It has been demonstrated that plasma MGO was able to discriminate between patients with both T1DM [9] and T2DM and healthy subjects [9]. However, MGO is very reactive, and therefore, the end-product of its metabolism, D-lactate, is often measured as a surrogate marker reflecting MGO concentration [16]. Nevertheless, it has been indicated that it would be worth elucidating to what degree D-lactate is the product of MGO conversion as opposed to the result of gut bacteria metabolism, because bacteria are also able to produce this compound [16]. The concentration of MGO metabolites has been increased several-fold in both insulin-dependent and non-insulin-dependent diabetes; however, there were differences between those two types of diabetes in respect to the glyoxalase system enzymes: while Glo1 activity has been upregulated in both types of diabetes, Glo2 exhibited elevation in non-insulin-dependent diabetes only [10]. This results in the accumulation of S-D-lactoylglutathione in the circulation of those with insulin-dependent diabetes and a negative correlation between D-lactate and GSH. Moreover, a positive correlation has been noted between the level of D-lactate and HbA1c [10].

Scheijen et al. [16], studying blood and urine samples of T2DM patients, also observed a positive correlation between D-lactate and HbA1c. The same correlation was found in the study conducted by Beisswenger et al. [17], although only in patients who were not treated with metformin. The administration of metformin, commonly used in diabetes treatment, eliminated the observed relationship. Additionally, T2DM patients treated with metformin had lower systemic levels of MGO and higher levels of D-lactate [17]. The authors proposed two possible explanations: either metformin binds α-dicarbonyl group of MGO or intensifies MGO detoxification through the glyoxylase pathway and, thus, increases the concentration of D-lactate [17].

Hyperglycemia leads to the increased formation of triosephosphate metabolites and MGO, but what is important, in the case of diabetic patients, is that these toxic metabolites are accumulating even at normal glucose levels [11]. It may be in part due to the fact that metabolism in diabetic subjects is faster. As a result, high concentrations of fructose-1,6-bisphosphate (FBP) are being produced, the compound, which after splitting, leads to the formation of glyceraldehyde-3-phosphate (GAP), which should be further processed by GAP dehydrogenase (GAPDH). Unfortunately, GAPDH could be downregulated by ROS, which can possibly lead to the elevation of GAP, which after conversion to DHAP, can be a source of MGO [11]. This hypothesis, however, requires further study.

Because diabetic patients are exposed to higher levels of MGO precursors, which also come from sources other than glucose, they are more susceptible to the development of diabetic complications, even if their glycemic status is under control [11,125].

Because D-lactate is a relatively stable end-product of MGO metabolism, Scheijen et al. [16] proposed that it could be considered as a possible indicator of diabetic complications.

Later, Schumacher et al. [81] reported the disturbances of an alternative pathway of MGO scavenging in diabetic patients. Whereas the components of the glyoxalase pathway, Glo1 activity and D-lactate concentration, did not discriminate between diabetic patients with and without complications, AKR activity and hydroxyacetone level showed the potential to do so. T2DM patients without complications had the highest concentration of hydroxyacetone and the greatest activity of MGO-dependent AKR in erythrocytes. As the authors suggest, in the case of advanced diabetes, it might be the minor pathway of MGO detoxification (leading via AKRs) that compensates for the faulty glyoxalase system. These findings are in agreement with the authors’ observations obtained in the *Glo1^−^*^/*−*^ murine model [81].

McLellan et al. [10] observed that the duration of diabetes positively correlated with the occurrence of diabetic complications, such as retinopathy, nephropathy, and neuropathy. They found elevated concentrations of MGO in the blood of diabetic patients and observed that the development of complications is the result of chronic exposure to high doses of this compound. They indicated that patients with such complications had higher ages, HbA1c concentrations, and Glo1 activity than patients without the complications. They concluded that patient’s age and duration of the disease are risk factors for the development of diabetic complications. The authors found HbA1c to be a risk factor for the development of diabetic complications and D-lactate as a risk factor for retinopathy. However, in the latter case, low D-lactate levels poses a higher risk, possibly due to the slow conversion of increased levels of MGO to D-lactate and hence, a longer and higher exposure.

Exposure to high glucose levels leads to the production of MGO and the intensification of MAGE production, which changes the properties of proteins. Tubular cells are responsible for the restoration of proteins and peptides, and this function is impaired in diabetes because, in hyperglycemia, tubular cells have a lower ability to handle RCS-modified proteins [126]. RCS-mediated nucleoside modifications have also been demonstrated in the kidneys of diabetic patients [73]. An elevated accumulation of CEdG has been noted in the kidneys of these patients, and it has been suggested that it may lead to the loss of genetic integrity in the kidneys of diabetic patients [73].

Chou et al. [15] observed that in the early stages of kidney damage, the level of D-lactate is elevated, while in the advanced stages it is declining. It reflected the fluctuations in MGO production. The authors suggested that in the early stages of renal dysfunction, self-reparatory mechanisms require huge energy input, and hence, the glycolytic flux is intensified. Later, when reparative processes are no longer possible, the fibrosis of tissue is progressing, and less energy is needed, hence there is lower MGO and subsequent D-lactate production [15].

Diabetic patients with neuropathy can suffer from pain and hyperalgesia. Glo1 activity in peripheral nerves is low, which leads to the accumulation of MGO [127,128]. Bierhaus et al. [9] observed increased concentrations of plasma MGO in patients with pain and that MGO can discriminate between diabetic patients with pain and without pain. They demonstrated that MGO causes the depolarization of sensory neurons and leads to changes in voltage-gated sodium channel Na*_v_*1.8. In a very interesting experiment, they subjected wild-type mice dorsal root ganglion neurons to the plasma of diabetic patients with and without pain and measured COX-2 as a surrogate marker of neural function. They observed that plasma from pain-suffering patients induced higher COX-2 transcription than plasma from patients without pain. It is interesting that the spiking of plasma from diabetic patients without pain with MGO caused COX-2 expression elevation. The mechanism by which MGO can influence channel function is so far unknown, and it is opening new research avenues connected to pain perception and therapeutic goals.

## 3. MGO and MAGEs in Cardiovascular Disorders

### 3.1. Pathological Routes Linking Metabolic Syndrome and Diabetes with Cardiovascular Complications

Pathological features characteristic of metabolic syndrome and diabetes (insulin resistance, hyperglycemia, hypertension, dyslipidemia, and obesity) increase the risk of cardiovascular disease (CVD) [129,130,131]. Micro- and macrovascular complications are typical for patients suffering from diabetes, and macrovascular pathologies mostly conditioned by atherosclerosis development yield cardiovascular diseases, including myocardial infarction [5,30,129,132,133]. Angiopathy, underlying these events, is associated with the dysfunction of vascular endothelium caused by oxidative stress, inflammatory processes, and ER stress, which impairs its vasodilatory functions, increases permeability, and enhances proatherogenic and prothrombotic features [131,133,134]. Both insulin resistance and hyperglycemia have been shown to decrease the generation of nitric oxide in endothelial cells and stimulate the production of plasminogen activator inhibitor-1 (PAI-1) [30], which would lead to impaired blood flow and hypertension, as well as disturbances in thrombolytic functions. Additionally, insulin resistance and hyperglycemia are implicated in dyslipidemia associated with an increase in lipoproteins/triacylglycerols, as well as free fatty acids in circulation [30]. For example, diabetic mice have been reported to show a decreased clearance of apo-B-48 lipoprotein remnants, which is associated with the dysfunction of extracellular matrix (ECM) components (perlecan HSPG), impairing the lipoproteins’ removal from circulation [135].

The mechanisms that link increased glycolytic flux with endothelial and, hence, cardiovascular impairment consider the stimulation of side pathways of glycolysis leading to the overproduction of sorbitol (polyol pathway), generation of glucosamine-6-phosphate (hexosamine pathway), and overproduction of trioses due to the inhibition of glyceraldehyde-3-phosphate dehydrogenase (GAPDH). Triose accumulation leads to the activation of protein kinase C (PKC) (by overproduced DAG), as well as MAGEs generation (due to the excessive generation of MGO) [5,30,131]. Sorbitol production catalyzed by aldose reductase is associated with the depletion of NADPH (exhausted in this reaction) and, hence, the decrease in GSH. Consequently, the polyol pathway would exacerbate the oxidative stress [30]. A hyperglycemia-induced hexosamine pathway leads to the generation of UDPGlcNAc molecules, whose accumulation enhances the binding of GlcNAc moieties to many proteins, which modifies their functions. For example, GlcNAc-glycosylated transcription factor Sp1 seems to induce the expression of the PAI-1 gene, whereas GlcNAc binding with eNOS impairs its activity. Such mechanisms result in PAI-1 increase and NO decrease [30]. Similar effects on PAI-1 and eNOS are observed upon PKC activation. Additionally, DAG-PKC-mediated signaling would upregulate endothelin-1 (ET-1), VEGF, TGF-β, NF-κB, and NADPH oxidases (NOXs). Such effects are associated with the induction of oxidative stress (via NOXs), inflammatory processes (NF-κB signaling), blood flow disturbances (eNOS decrease and ET-1 increase), angiogenesis and vascular permeability (VEGF), as well the occlusion of blood vessels resulting from an impairment of fibrinolysis (PAI-1 impact) and an excessive production of ECM components (type IV collagen and fibronectin possibly upregulated by TGF-β) [30,136]. Triose-derived dicarbonyl molecules (mainly MGO) show much greater efficiency in AGE formation in comparison to glucose [30]. Therefore, they modify intra- and extracellular proteins, the latter being components both of ECM and plasma proteins. These actions impair the functionality of blood vessels, e.g., via decreasing their elasticity through the disturbances in collagen’s structure or stimulating prooxidative, proinflammatory, and procoagulatory processes (induced by blood plasma AGEs binding with their receptors on macrophages or endothelial cells) [30]. The upregulation of AGEs receptors (RAGEs) and their ligands being observed under hyperglycemic conditions further adds to ROS and inflammation enhancement, contributing to vascular endothelium destruction [137].

**Figure 2:**
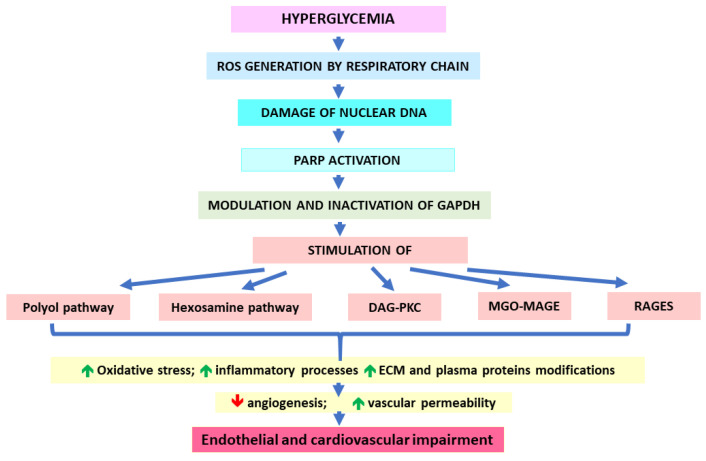
Impact of hyperglycemia on cardiovascular complication development.

As proposed by Brownlee and Giacco [30,132,137], all five pathological pathways mentioned above (polyol, hexosamine, DAG-PKC, MGO-MAGEs, and RAGEs) are initiated by the generation of hyperglycemia-induced ROS by the mitochondrial respiratory chain. Enhanced reactive oxygen species would damage nuclear DNA, which in turn, would activate PARP. Subsequently, active PARP would modify GAPDH via ADP-ribosylation (using NAD^+^). This would lead to the inhibition of GAPDH and the obstruction of glycolytic pathway at triose level. Consequently, the accumulation of the above-mentioned side products and their detrimental effects are observed (Figure 2). Because most of these pathological pathways further stimulate ROS generation, the auto-augmentation of such routes would deepen the metabolic disturbances in the vicious circle mode. This mechanism seems to be similar in the case of both micro- and macrovascular complications. However, the causative relationship between hyperglycemia and cardiovascular disorders is not so obvious as in the case of microvascular complications [72]. Actually, CVDs seem to be more conditioned by insulin resistance, which (due to the lack of insulin-mediated inhibition) stimulates the release of free fatty acids (FFAs) from the adipose tissue. These FFAs are taken up by endothelial cells and undergo (uncontrolled by insulin) excessive β-oxidation yielding the substrates for the respiratory chain. Consequently, as in the case of hyperglycemia-conditioned accelerated aerobic glycolysis, excessive ROS production is observed, which triggers most of the pathological pathways discussed earlier [137,138].

### 3.2. MGO/MAGEs Contribution to Blood Vessels Wall Impairment, Hypertension, Dyslipidemia and Atherosclerosis

#### 3.2.1. Blood Vessels Focusing on Endothelium—Impairment of Angiogenesis

In light of the above-mentioned mechanism, MGO and its glycation end products comprise an important causative pathway contributing to vascular pathologies conditioned by hyperglycemia, hyperlipidemia, and insulin resistance [133]. AGEs are involved in the induction of oxidative stress in the endothelial progenitor cells (EPCs), as well as the downregulation of antioxidative and anti-inflammatory enzymes (catalase, superoxide dismutase, and paraoxonase 2) and eNOS, but the upregulation of NOXs in human endothelial cells, which impairs the function and healing of endothelium [133,139,140,141]. Increased glycolytic/FFAs flux in mammalian endothelial cells has been shown to elevate ROS generation, which in turn, raises MGO and MAGEs levels [30,132,137,138,139,142]. On the other hand, Glo1 upregulation attenuates these effects [142,143]. For example, high-Glc exposed Glo1-knockdown human aortic endothelial cells (HAECs) have shown an increase in MGO, followed by the upregulation of inflammatory processes, endothelial dysfunction, and disturbances in ECM components [144]. Additionally, MGO treatment of HAECs has induced ROS generation [145], as well as the cell apoptosis associated with oxidative stress connected (at least partially) with the impairment of the antioxidative thioredoxin/peroxiredoxin system [146]. The effects leading to oxidative stress development might be associated with/augmented by NOS activation, as observed by Miyazawa et al. [145]. However, in other studies, increased Glc/MGO demonstrated no effect on eNOS in HAECs [144], in accordance with the lack of eNOS inhibition by MG-H1 and AP observed in other experiments on the human endothelium [147]. Unlike in HAECs, in human umbilical vein endothelial cells (HUVECs), MGO exposure led to the inhibition of eNOS activity and NO production, probably via the attenuation of eNOS Ser-1177 phosphorylation and Akt phosphorylation [139,148]. In turn, ROS accumulation was observed in MGO-treated HUVEC cells, probably as a consequence of NOX upregulation and/or SOD-1/CAT/GPX downregulation [102,139,148]. Similarly, in human endothelial EA.hy926 cells, MGO treatment caused a decrease in eNOS Ser-1177 phosphorylation associated with the uncoupling of this enzyme and superoxide radical generation [149]. Signaling pathways engaged in MGO-mediated vascular impairment associated with ROS generation and the mitochondrial-dependent apoptosis of endothelial cells, have been proposed by Wang et al. [102]. In their experiments on MGO-treated HUVECs and mice, the authors reported the involvement of PI3K/Akt/Nrf2/HO-1 routes, which upon MGO inhibition, led to the downregulation of antioxidative enzymes and the upregulation of oxidative stress coupled with mitochondrial dysfunction, as well as proapoptotic and proinflammatory events. These pathological routes were attenuated by metformin both in the endothelial cells and in mice [102] (Table 1). Similarly, in other experiments on HUVECs, MGO treatment has induced mitochondrial-dependent apoptosis, the impairment of the Akt/eNOS/NO pathway, and the upregulation of prooxidative (NOX4/ROS) and proinflammatory (NF-κB) routes, all of which were reversed by phosphocreatine and NAC [150].

Many MGO-affected routes may be mediated by its impact on p53 protein, which is induced in response to cellular stress [151,152]. Upon the accumulation of DNA damages, p53 inhibits the cell cycle diverting the cell toward apoptosis. However, p53 also controls a variety of “non-classical” pathways, such as metabolic homeostasis, ferroptosis, autophagy, and senescence [153]. MGO has been shown to alter the genomic profile associated with cell cycle regulation, especially the p53 pathway [151] in HUVECs. Additionally, MGO exhibited the stimulatory effect on p53 signaling in the same type of HUVEC cell line, in which MGO caused DNA damage and induced p53 phosphorylation associated with the inhibition of mTORC1 and the stimulation of autophagy [152]. Moreover, a prolonged MGO exposure can divert endothelial cells into senescence phenotype, as has been reported in human vascular endothelial cells (HVECs) from patients suffering from coronary heart disease. HVECs’ exposure to combined MGO and GO action led to the cells’ senescence through the increase in ROS and upregulation of p21 [154].

In search of other signaling pathways presumably engaged by MGO/MAGE, human aortic endothelial cells (HAECs) derived from healthy and T2DM individuals have been examined. In comparison with untreated healthy cells, both diabetic and MGO-exposed healthy cells were dysfunctional and showed upregulation of three MAPK pathways. Additionally, it seems that the MGO induction of the K_ATP_ channel contributed partially to MGO-caused deleterious effects on endothelial cells (via the JNK pathway) [155].

These and other experiments conducted on endothelial or endothelial progenitor cells (EPCs) exposed to MGO suggest its causative effect on endothelium dysfunction [67,139,148,150,156,157,158,159]. As mentioned above, prooxidative and proinflammatory pathways induced by MGO can also affect angiogenesis process. For example, MGO-treated murine EPCs [158], as well as human and bovine endothelial cells [156] have demonstrated decreased VEGFR-2 levels, probably mediated by MAGEs’ induction of RAGE. This was associated with the impaired capability of blood vessel tube formation [156,158]. Hence, MGO seems to impair the angiogenesis process, which might be partially corrected by Glo1 overexpression, as has been shown in diabetic rats [160]. In endothelial cells, the mechanism responsible for MGO/RAGE-induced VEGFR-2 degradation (leading to a decrease in tube formation) was the peroxynitrite (ONOO^−^)-mediated autophagy process (this finding was supported in diabetic mice aortas experiments) [156]. Therefore, the authors suggested that the mechanism leads to a decrease in angiogenesis, which starts from hyperglycemia associated with MGO increase. Further, MGO induces RAGE, which leads to peroxynitrite generation and VEGFR-2 degradation through the autophagic pathway, with the final result in lowered angiogenesis. Another route associated with MGO-impaired angiogenesis has been proposed by Nigro et al. [161]. In their in vitro and in vivo experiments, the authors observed the involvement of NF-κB-p65 and HoxA5 in MGO-stimulated effects leading to the downregulation of VEGFR-2 and the decreased capability of new vessel formation in both MAECs from Glo1KO mice and MGO-stimulated MCECs [161]. Both NF-κB-p65 and HoxA5 were also upregulated in high-fat diet-fed mice that developed diabetic characteristics. Judging from these observations, it might be suggested that MGO induces NF-κB-p65, which further binds with the promoter region of HoxA5, activating this transcription factor. In turn, HoxA5 stimulates signaling pathways preventing angiogenesis [161]. In line with these findings, Glo1 overexpression in bone marrow-derived circulating angiogenic cells extracted from diabetic mice has demonstrated protective actions on blood vessels, restoring the cells’ viability and potential toward neovascularization (impaired under hyperglycemic/hypoxic conditions) [162]. Conversely, Glo1 silencing in human endothelial cells has caused the upregulation of proinflammatory and pro-adhesive factors [163].

MGO/MAGE-weakened vasodilatory and/or angiogenic capacity both in endothelial and EPC cells would enhance the risk of CVD in diabetic patients. Particularly, EPCs circulating in the blood plasma play important functions in repairing blood vessels, hence, their impairment may diminish the healing forces of the organism with respect to the cardiovascular system.

#### 3.2.2. Cardiovascular System in Animal Models

MGO treatment of both normal (Wistar and Sprague Dawley rats) and diabetic rats (GK rats) has impaired or worsened the condition of the animals’ cutaneous vasculature or aortas, namely, their vasodilatory functions or increased cardiac fibrosis, which was accompanied by the deterioration of oxidative status, inflammation, and glycation [96,98,105,139] (Table 1). Similarly, vasorelaxation has been impaired by diabetes and/or Glc/MGO treatment in mesenteric arteries (from STZ-treated or normal Wistar rats) [163,164,165]. This effect was accompanied by the intracellular elevation of MG-H1, CML, VCAM-1, ICAM-1, and nitrosative stress and was corrected by antioxidants and/or Glo1 overexpression [163,165]. The MGO increase in murine models (MGO-fed or Glo1 inhibitor-treated apoE KO mice) to the level characteristic for diabetes has enhanced vascular adhesive properties, as well as atherogenesis. Those effects were comparable to the diabetic ones, although the mice had normal glucose levels [166]. Some of the effects leading to increased adhesiveness and inflammation were RAGE-independent (observed in RAGE-deficient mice); however, others required the presence of RAGE (e.g., MCP-1 upregulation) [166]. In C57BL/6 mice, MGO treatment impaired oxidative status (through a decrease in antioxidative enzymes and an increase in the lipid peroxidation marker) and increased the cytokine level in circulation. This was associated with disturbances in aorta structure reflected by its increased thickness and apoptosis level and downregulation of Akt/Nrf2 route in the aorta (as simultaneously observed in HUVECs) [102]. Metformin pretreatment attenuated most of these deleterious MGO effects both in the mice and human endothelial cells (HUVECs) [102] (Table 1). In STZ-treated rats, diabetes lowered Glo1 activity and upregulated some AGE markers (3-deoxyglucosone (3-DG) and CML) but had no impact on MGO and CEL levels in the rats’ hearts [167]. Additionally, diabetes altered the expression of genes associated with oxidative stress, DNA damage, heart fibrosis, and inflammation, which was partially attenuated by Glo1 overexpression [167]. A protective effect of Glo1 overexpression against diabetes-induced cardiovascular impairment has been demonstrated in diabetic mice, in which it improved vascular inflammation and heart muscle condition [157]. However, Glo1 overexpression has not guarded against diabetes-associated atherosclerosis or endothelial dysfunction [168,169], neither has Glo1 under-expression enhanced atherosclerosis in a murine model [106] (Table 1). Although Glo1 expression manipulation was expectedly correlated with the overall aortal MG-H1 level, it did not show any impact on the degree of aortal atherosclerotic lesions or aortal collagen glycation (including MG-H1) [168,169]. Nevertheless, because these studies were conducted on apoE-deficient mice models, it might be suggested that characteristic for them, dyslipidemia may have interfered with the obtained results. For example, as the authors presumed, intracellularly working Glo1 metabolizes mainly glycolysis-derived MGO, whereas under conditions of extracellular lipid overload accompanied by inflammatory/oxidative stress, it may be lipid peroxidation that is the additional source of MGO/MAGE not handled by Glo1, and thus, enhancing pathological routes [169]. A causative input of MGO/(M)AGE in the development of atherosclerosis in a diabetes murine model accompanied by human endothelial cell experiments has been observed by Fang et al. [148]. The authors reported elevated levels of MGO and protein carbonyls in the aortas of diabetic ApoE-deficient mice, in which atherosclerotic lesions were increased. These effects were associated with increased ROS, the downregulation of antioxidative enzymes, as well as the decrease of Akt and eNOS phosphorylation and the decrease in aortal GSH and serum nitric oxide. Because all of these pathologies were attenuated by NAC, it might be supposed that NAC-enhancing GSH synthesis would contribute to the more efficient scavenging of MGO by the glyoxalases system [148].

#### 3.2.3. Cardiovascular Disorders in Patients

As previously discussed, MGO and its metabolic and glycation end products are elevated in diabetic individuals. Enhanced (M)AGEs levels have been observed in blood plasma/serum both in T1DM [21] and T2DM patients [22,23], and in T2DM individuals, MG-H1 concentration was increased, especially in retinopathy cases [24]. AGEs have been associated with a higher risk of the incidence of cardiovascular events in both T2DM (CEL and CML) [23] and T1DM (CEL, CML, and pentosidine) [170]. However, no independent association was observed between AGEs (CEL, CML, and pentosidine) and prior cardiovascular events in diabetic (T2DM) [23,171] and nondiabetic individuals [171], which might be connected with the AGE-scavenging effects of medicines applied in CVD treatment (as discussed in Chapter 4).

When CML concentrations were estimated in association with CVD mortality risk in older individuals, such a connection was observed in a nondiabetic subpopulation in which higher blood serum CML levels raised the risk of dying, especially of CVD [172,173]. In addition to CML, in older nondiabetic women, increased soluble RAGE forms (sRAGE and esRAGE) were connected with higher CVD-caused mortality rates [173]. In line with these findings, in an 18-year follow-up study comparing the association of MG-H1 with CVD mortality in diabetic and nondiabetic cohorts, only nondiabetic women showed a positive correlation; those who died of CVD had higher MG-H1 serum levels [174,175]. Other studies have shown no association or even inverse dependance between AGE/CML and CVD events/mortality [176,177]. In T2DM individuals with nephropathy, serum CML levels were not correlated with cardiovascular events [176], whereas in hemodialysis patients, low CML levels were connected with higher all-cause mortality rates and showed a tendency toward greater CVD mortality [177].

Although the above observations seem to be inconsistent, showing either the association of AGEs with CVD or the lack of such a dependance, it is probably connected with differences in studied populations and applied methodology (Ab-based ELISA vs. chromatography/mass spectrometry, as well as statistical approach, as discussed by Hanssen et al. [23]). Therefore, stronger evidence speaks in favor of greater CVD risk in individuals with higher (M)AGEs, not only in older people but also in diabetics. Similar uncertainty exists regarding blood plasma MGO level association with CVD occurrence. Hanssen et al. reported a positive association between a higher fasting MGO level in plasma and the increased risk of the incidence of CVD, both in T1DM [178] and T2DM [179] patients. Also, in T2DM individuals, blood plasma MGO has been estimated as a predictor of intima media thickening, vascular stiffening, and blood pressure elevation [180]. However, in their other study, Hanssen et al. [181] reported no correlation between higher fasting and post-OGTT plasma MGO levels and prior CVD in cohorts with normal, prediabetic, and diabetic (T2DM) conditions. Therefore, supposedly unlike macrovascular complications, microvascular complications (CKD and retinopathy) seem to be connected with elevated MGO [181].

#### 3.2.4. Atherosclerosis

(M)AGEs have been detected in atherosclerotic plaques and atherosclerotic-like lesions [21,56,182,183]. CML accumulation has been observed in macrophages and at calcification sites in degenerated aortic valves [183]; CML colocalization with THP has also been found in macrophages within atherosclerotic plaques extracted from coronary arteries derived from control and diabetic individuals [21]. Additionally, THP which is an MGO-derived AGE, was elevated in T1DM patients’ serum and positively associated with soluble vascular cell adhesion molecule 1 (sVCAM-1) (unlike other AGEs: CML, CEL, and pentosidine) and secreted phospholipase A 2 (sPLA2) [21]. Because elevated sVCAM-1 and sPLA2 have been associated with atherosclerotic processes running in the organism [184,185], THP might be involved in CVD development.

When (M)AGEs have been estimated in human carotid endarterectomy specimens, higher levels of CML and MG-H1 were observed in rupture-prone plaques (in comparison with stable ones). CML and MG-H1 were localized mainly in macrophages around the necrotic core (but also in endothelial cells), and they were correlated with inflammatory (IL-8, MCP-1) and proapoptotic (cleaved caspase 3) markers, as well as matrix metalloproteinases (MMP-9) [182]. These findings were accompanied by lowered Glo1 mRNA/protein in ruptured plaques (present in all cells of the plaque, except for the necrotic core), but no change in RAGE expression was observed. Also, no associations were found between the studied AGEs and plasma glucose levels or between plaques coming from diabetic vs. nondiabetic patients. In search of the cause-and-effect relationship between MGO/MAGE and inflammation, the authors performed experiments on human monocytes. They observed that although TNF/hypoxia decreased Glo1 and upregulated MGO/MAGEs, MGO/MAGEs did not mediate the TNF-induced secretion of IL-8, MCP-1, and MMP-9. Nevertheless, Glo1 knockdown worsened the viability of MGO-exposed cells, which suggests a Glo1 protective function in this system [182]. The lack of MGO engagement in proinflammatory pathway stimulation has also been observed in TNF-α induced HUVECs, in which MGO led to a decrease in VCAM-1 [151].

In light of the above data, MGO/MAGE seem to be involved in the processes accelerating atherogenesis. However, the pathological events mediated by α-dicarbonyl compounds and their glycation end products occurring in the blood vessels and atherosclerotic plaques are not necessarily reflected by their levels in the blood plasma or serum, as has been recently reported by Berge et al. [186]. In search of prognostic markers that could be applied in the evaluation of the coronary artery disease (CAD) risk conditioned by coronary atherosclerosis in middle-aged or older male athletes, the authors found no associations between the plasma concentrations of α-dicarbonyl compounds (MGO, GO, 3-DG) or (M)AGEs (CML, CEL, MG-H1) and the number and type of coronary artery plaques or the level of coronary arteries calcification. However, as reported in an earlier study, serum CML level was elevated in CAD patients (especially in diabetics), but it was pentosidine that was correlated with CAD [187]. The causative involvement of MGO/MAGE in CAD has been suggested in an integrative genomics study, in which the *GLO1* gene was found to be associated with CAD pathology [188].

#### 3.2.5. Endoplasmic Reticulum Stress (ER Stress) Followed by Unfolded Protein Response (UPR) in Blood Vessels

MGO intracellular accumulation upon hyperglycemia and associated cardiometabolic disturbances leads to the modification of multiple proteins, which impairs proper protein folding and trafficking. As a consequence, endoplasmic reticulum stress (ER stress) may develop, which can further induce unfolded protein response (UPR) to restore protein homeostasis. UPR can trigger three pathways mediated by protein kinase RNA-like ER kinase (PERK), inositol-requiring protein 1α (IRE1α), and transcription factor 6 (ATF6). However, when pathological changes exceed the capacity of UPR, harmful pathways are accelerated, leading to oxidative stress, inflammation, and cell death via apoptosis. As recently reviewed by Ren et al. [189], ER stress with UPR and other route initiation leading to such deleterious effects is associated with cardiovascular disease. MGO involvement in these processes has been shown in endothelial (HAECs) and vascular smooth muscle cells (VSMCs). Glc-treatment (or Glo1 silencing) of HAECs led to the accumulation of MGO/MAGE in the cells, as well as the upregulation of UPR pathways associated with an increase in heat shock proteins and the stimulation of proinflammatory and prothrombotic routes. The protective effects of *trans*-resveratrol and hesperetin combination (tRES-HESP) was shown in this model; tRES-HESP increased the expression of Glo1 and decreased the expression of hexokinase-2, in this way, correcting Glc metabolism and diminishing MGO level/effects [190]. Similarly, experiments on MGO-treated rat aortal VSMCs indicated MGO involvement in the induction of ER stress because MGO caused the upregulation of three UPR pathways (PERK, IRE1α, and ATF6). However, no apoptosis was triggered in MGO-treated VSMCs [191].

#### 3.2.6. Hypertensive and Procoagulatory Properties of MGO/MAGE

Hypertension, being one of the components of metabolic syndrome, is the leading risk factor for CVD [192]. MGO and MAGE involvement in hypertension has been addressed in experiments conducted on spontaneously hypertensive rats (SHR), which develop genetically conditioned hypertension, which is not associated with insulin resistance or hyperglycemia [193,194]. Elevated levels of MGO in blood plasma, the aorta, the liver, and the kidneys (but not heart) and (M)AGEs in the aorta, mesenteric artery, and kidneys have been demonstrated in these animals [108,109,110,111]. These effects were accompanied by oxidative stress indicated by the increase in superoxide radical and hydrogen peroxide and the decrease in GSH or GSH/GSSG ratio [108,109,110] (Table 1). Similar findings have been reported in Sprague Dawley (SD) rats, which, due to being fructose-fed or MGO-treated, developed hypertension associated with MGO upregulation and the initiation of prohypertensive routes [31,94,104] (Table 1).

Additionally, the MGO/MAGE-mediated dysfunction of blood vessels reflected by morphological changes in mesenteric arteries was shown. This was associated with eNOS downregulation, eutrophic inward vascular remodeling, and the impairment of endothelium-dependent relaxation in hypertensive rats [31,110,111] (Table 1). These disturbances seem to be mediated by MGO/(M)AGE-triggered oxidative and/or (NF-κB-mediated) inflammatory pathways, as has been demonstrated in SHR-derived VSMCs [195] and rats’ VSMCs exposed to Fru or MGO [104,196]. The pathological events connected with hypertension, MGO/(M)AGE upregulation, oxidative stress, and blood vessel impairment have been corrected by MGO/(M)AGEs scavengers: aminoguanidine [110,111], metformin [31], or alagebrium [104]. Moreover, the proapoptotic MGO effect on HUVECs has been inhibited by telmisartan (a selective angiotensin II type 1 receptor (AT1R) blocker) [197]. These observations indicated MGO/(M)AGE being involved in the development of genetically conditioned hypertension (independent of hyperglycemia/diabetes), as well as hypertension caused by dietary fructose/glucose overload. The mechanism underlying MGO-induced hypertension probably involves the activation of the RAAS system through the MGO/RAGE/NF-κB route [104] (Table 1). Additionally, the MGO-induced activation of aortal smooth muscle cell proliferation might contribute to vascular impairment and hypertension, as suggested by Chang et al. [95]. In their experiments on Fru or MGO-treated rats, as well as aorta-derived vascular smooth muscle cells (VSMC), they observed the MGO-stimulated proliferation of VSMC, the effect of which was mediated by the MGO activation of Akt1. Namely, MGO was shown to form an adduct with the Cys-77 residue at Akt1, probably changing the protein conformation. This, in turn, led to Akt1 activation via Ser-473 phosphorylation yielding the cell proliferation [95] (Table 1).

On the other hand, a short-term MGO exposure to the aorta and mesenteric artery (devoid of endothelial layer) has exerted an inhibitory effect on the noradrenalin-induced contraction of VSMCs [198]. This seemed to have been mediated by MGO opening one type of calcium-activated potassium channel [198].

Disbalance between blood coagulation cascade and the thrombolytic system, which promotes thrombosis, increases the risk of cardiovascular incidents associated with blood vessel occlusion, such as myocardial infarction. MGO has been shown to be able to form adducts with antithrombin III (ATIII). MGO modification of ATIII at Arg 393 led to ATIII inhibition reflected by its inefficient blocking of thrombin and factor Xa [199]. Hence, MGO might contribute to the impairment of the processes responsible for the inhibition of thrombus formation, and in this way, enhance the risk of CVD, especially in patients with metabolic syndrome/diabetes characterized by an elevated plasma MGO level.

#### 3.2.7. Dyslipidemia

Qualitative and quantitative disturbances in lipids and lipoproteins in circulation (observed in metabolic syndrome) are strongly associated with the induction and development of CVD. Pathologically altered LDL particles, such as oxidized LDL (oxLDL) and small dense LDL (sdLDL), are particularly involved in the process of atherogenesis [200]. MGO seems to play an important role in LDL modifications, making them more prone to accumulate in blood vessel walls, which is associated with the induction of proinflammatory events, the formation of foam cells, and hence, atherosclerotic plaque development. MGO involvement in LDL particle alteration has been demonstrated by Rabbani et al. [201,202]. The authors observed enhanced LDL apoB100 glycation at Arg and Lys residues in T2DM patients [202]. Increased apoB100 AGEs included MGO-derived MG-H1, CEL, and MOLD, as well as other α-dicarbonyl-derived AGEs (G-H1, 3DG-H, and pentosidine). Minimally MGO-modified LDL particles tended to change their features; their size dropped (resembling sdLDL), and they showed greater binding to proteoglycans associated with atherosclerotic plaques (biglycan, aggrecan, and perlecan) [201]. Additionally, the authors reported that Arg18 modified by MGO leads to a conformational change in apoB100, which enhances LDL binding with proteoglycans. Thus, minimally MGO-modified LDL particles increased adhesiveness to the aorta wall through binding with heparan sulfate-containing proteoglycans [201]. Therefore, it is probably MG-H1-altered Arg18 on apoB100 that changes LDL particles’ properties, enhancing their binding with blood vessels’ proteoglycans containing heparan sulfate. This, in turn, would extend the time of LDL particles’ attachment to the endothelium and their exposure to RONS/α-dicarbonyl stressors, further enabling the modifications of LDL particles toward more proatherogenic characteristics [201,202]. However, minimal MGO modification did not alter LDLs’ affinity for their receptors on hepatocyte-like cells and fibroblast and did not make them recognizable by scavenger receptors on macrophages. Additionally, their clearance from the murine organism was not changed [201]. Besides apoprotein modification, MGO may also take part in lipid oxidation, as has been demonstrated by Lankin et al. [203], who reported MGO contribution to LDL lipoperoxidation (mediated by ROS generation) under hyperglycemic conditions. Metformin treatment for diabetic patients has been shown to inhibit MGO-mediated LDLs modifications [202,203].

Similarly, MGO and other dicarbonyls seem to modify HDL particles, exacerbating their cholesterol scavenging and antioxidative and antiatherogenic functions, especially in diabetics [204,205]. MGO-derived MG-H1 in HDL particles mainly seems to modify Arg residues in apolipoprotein A1 (apoA1), which alters its conformation. The observed consequences include the conversion of HDL particles into smaller and denser particles, which tend to be easier to remove from circulation, hence, their concentration falls [103,204]. Additionally, HDL-mediated reverse cholesterol transport, as well as this lipoprotein’s protective actions may be impaired, such as cholesterol esterification (through weakened LCAT binding), cholesteryl esters exchanging for TAGs (via CETP inhibition), and antioxidative/anti-poisonous properties (weakened PON1 binding) [204].

Therefore, MAGEs seem to participate in the conversion of both LDL and HDL particles into proatherogenic ones, mainly contributing to the impairment of the functionality of their apoproteins.

Generally, as recently discussed by Schalkwijk et al. [133], whereas the involvement of AGEs in the pathomechanism of cardiovascular complications in diabetes is well known, less scientific data are available that explain the exact function of MGO. MGO, being the major precursor of AGEs, contributes to endothelial dysfunction through the induction of oxidative stress, inflammation, ER stress, and apoptosis. Together with AGEs, it impairs angiogenesis and promotes atherosclerosis-associated inflammation. However, its role in AGE-stimulated cholesterol accumulation in macrophages, the phenotypic switch of vascular smooth muscle cells (VSMCs) into a macrophage-like state, and VSMCs calcification is not elucidated [133].

## 4. Potential Glycation Inhibitors and MGO Scavengers—Therapeutic Strategies

A lot of therapeutic strategies have been investigated in search of the attenuation of AGE-mediated prooxidative and proinflammatory effects associated with cardiometabolic diseases. For example, the beneficial actions of polyphenols were addressed recently by Dong et al. [206], who described different mechanisms in which polyphenols counteract AGE-RAGE-induced proinflammatory routes or switch the signaling pathways from AGE-stimulated proapoptotic events into autophagic routes, thus saving the cells.

**Figure 3:**
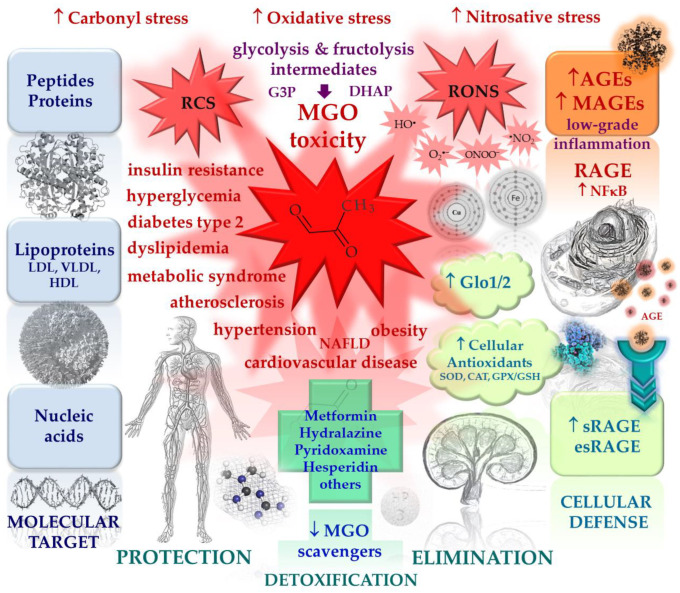
The main actors in the process of nonenzymatic glycation, cellular defense mechanisms against excessive glycation and accumulation of MGO and MAGEs, as well as known glycation inhibitors and MGO scavengers. Red arrows indicate pathological processes, blue arrows indicate protective mechanisms; ↑ increase/activation, ↓ decrease/inhibition.

**Figure 4:**
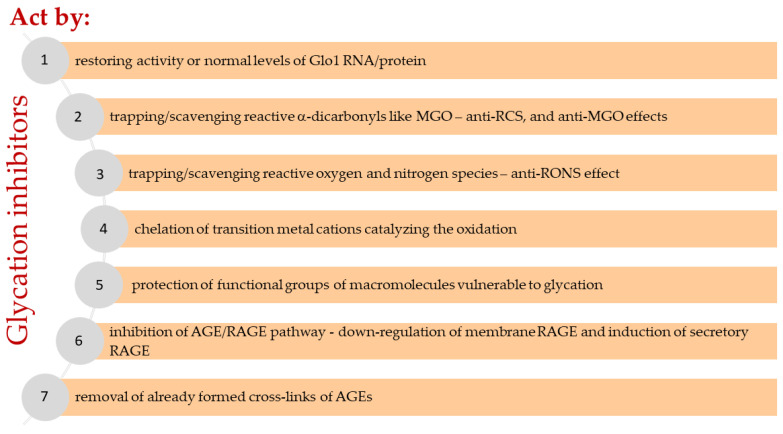
Assumed mechanisms of glycation inhibitors and related pathways.

Several therapeutic approaches are considered to reduce or prevent MGO-induced toxicity (Figure 3 and Figure 4). The first is an activation of the glyoxalase system (Glo1/2). However, this approach is limited to conditions in which the amount of enzyme or glutathione is insufficient. Unfortunately, we currently do not know of many inducers of glyoxalase. The reduced activity of Glo1 is restored, for example, by candesartan [207,208] and pyridoxamine [209]. The scientific literature also more extensively describes this effect for a combination of two plant polyphenols *trans*-resveratrol, and hesperetin (tRES-HESP, a combination of stilbenoid and flavonoid, respectively) [210,211]. However, no effect on Glo1 was confirmed for the other flavonoid isoquercitrin (=quercetin-3-*O*-glucoside) [212]. Nevertheless, glycation inhibitors are usually characterized by more than one mechanism of action determining the overall anti-AGE potential. Further strategies assume that highly reactive MGO can be neutralized by mechanisms independent of the glyoxalase system, such as uptake (chemical binding) using small molecules, contributing to its removal from the extracellular and intracellular environment. The uptake of MGO in statu nascendi is thought to prevent its direct toxicity but also to reduce glucotoxicity induced by ROS and RNS (together known as RONS), the formation of AGEs, and the interaction of AGEs with the transmembrane receptor, RAGE. The binding of AGEs to RAGE on the surface of cells, including immune, endothelial, and vascular smooth muscle cells or platelets, induces an intracellular response to carbonyl stress, oxidative stress, and nitrosative stress, characterized by the activation of transcription factors such as NF-κB. Vascular wall stress induced by RCS and RONS is characteristic of atherosclerosis, and the resulting activation of NF-κB and MAPK systems may be a possible mechanism for AGE-induced angiopathy [213,214,215]. The excessive production of RONS generates a flux of reactive carbonyls, whose accumulation is positively correlated with the development of autophagy [215]. In this context, a strategy oriented toward the molecular cascade of the AGE-RAGE axis, or more accurately the MAGE-RAGE axis, opens up a new pharmacological approach [216,217].

In general, molecular mechanisms for inhibiting excessive nonenzymatic glycation include any biological or chemical reaction that can reduce or prevent the generation of glycated macromolecules (peptides, proteins, lipoproteins, and nucleic acids) in vivo to reduce the formation of AGEs and interrupt the sequence of adverse events resulting from their deposition and leading to cell, tissue, and organ dysfunction. These reactions can occur both intracellularly and extracellularly, possibly simultaneously at multiple sites. Seven molecular pathways (Figure 4) and related mechanisms have been proposed by which low molecular-weight compounds known as glycation inhibitors reduce the levels of RCS and AGEs (mainly MGO and MAGEs) in the body:

(1) Restoring activity or normal levels of Glo1 RNA/protein.

(2) Trapping/scavenging reactive dicarbonyls (anti-RCS, anti-MGO, etc.), for example, methylglyoxal, glyoxal, malonyl dialdehyde, or others from both carbohydrate and lipid and some amino acid (threonine) metabolism, resulting lowered carbonyl stress.

(3) Trapping/scavenging reactive oxygen and nitrogen species (RONS) yielding direct or indirect antioxidant effect (e.g., by quenching radicals, earlier termination of radical reactions), as well as upregulating the antioxidant protection system (superoxide dismutase, catalase, glutathione peroxidase, glutathione, etc.) and downregulating prooxidative enzymes (e.g., NOX and iNOS), leading to a reduction in oxidative stress and nitrosative stress.

(4) Chelation of transition metal cations catalyzing the oxidation of monosaccharides, fatty acids, cholesterol, amino acids, nucleotides, and secondary reactions of glycated macromolecules.

(5) Protection of functional groups of macromolecules vulnerable to nonenzymatic glycation, e.g., by reversible noncovalent binding to functional groups of intra- and extracellular components subject to the glycation reaction or oxidative transformation (for example, serum albumin, lens crystalline, collagen, or Amadori products). Various interactions, such as hydrogen bonds formation, electrostatic forces, and hydrophobic and polar interactions, are usually responsible for such interactions.

(6) Inhibition of AGE/RAGE pathway—the downregulation of membrane RAGE (RAGE antagonists) and induction of secretory RAGE represent attractive targets for treating pathogenic glycation-related diseases. The interaction of AGEs with transmembrane RAGE results in the activation of proinflammatory genes (mediated by NF-ĸB) and ROS generation; hence, it is involved in the pathophysiology of many age-associated diseases, including cardiovascular disease. Some isoforms of this receptor lack a transmembrane domain and are, therefore, secreted from cells as sRAGE (soluble RAGE produced by alternative splicing) or esRAGE (endogenous secretory RAGE proteolytically exfoliated by metalloproteinases). The most circulating RAGE comes from sRAGE, and esRAGE accounts for only a minor fraction (about 20%). Circulating sRAGE and esRAGE compete with RAGE for ligand binding, and they act as a decoy to eliminate existing AGEs. Thus, increasing the level of circulating RAGE can reduce the activation of the AGE/RAGE pathway [218].

(7) Breaking AGEs cross-links—the mechanism attributed to the action of alagebrium (ALT-711).

Mechanisms (1) to (5) essentially prevent and limit the accumulation of AGEs and their precursors (MAGEs and MGO) in biological systems, pathway (6) inhibits RAGE-mediated signaling. On the other hand, mechanism (7) offers the removal of already formed AGE cross-links, which would be suitable for reducing existing cellular and tissue burdens associated with protein cross-linking, such as blood vessel wall stiffness contributing to hypertension. Results from clinical trials indicate that alagebrium may be the first representative of agents with this specific action in diseases associated with AGE accumulation [219].

MGO (methylglyoxal) is generated as a result of the spontaneous fragmentation of two intermediates of glycolysis and fructolysis: G3P (glyceraldehyde 3-phosphate) and DHAP (dihydroxyacetone phosphate). Additionally, its minor quantities can be derived from the metabolism of acetone, aminoacetone, and threonine, as well as from highly processed foods characterized by high content of glucose and fructose. The excessive consumption of simple sugars has been associated with the development of insulin resistance, hyperglycemia (enhanced by gluconeogenesis), and dyslipidemia (enhanced by de novo lipogenesis resulting in an increased level of free fatty acids, as well as hypercholesterolemia and hypertriglyceridemia). These metabolic disturbances lead to disorders such as NAFLD (nonalcoholic fatty liver disease), obesity, metabolic syndrome, and T2DM (diabetes type 2). Hyperglycemia observed in diabetics enhances the metabolism of glucose, accelerating the generation of MGO and its advanced glycation end products—(M)AGEs. Moreover, stimulated aerobic glycolysis increases reactive oxygen species and reactive nitrogen species (RONS) production (induces oxidative stress and nitrosative stress), which leads to the inhibition of glyceraldehyde 3-phosphate dehydrogenase (GAPDH). Finally, because glycolysis and fructolysis are inhibited at the level of trioses, more G3P and DHAP are produced, resulting in more MGO (carbonyl stress). Thus, considering MGO-caused damage to blood vessels, T2DM patients show an increased risk for cardiovascular complications (angiopathy and cardiomyopathy), which might result from the inefficient control of postprandial glycemia. Increased in diabetes, MGO and (M)AGEs show a deleterious impact on endothelium function, which is associated with the promotion of oxidative stress, low-grade inflammation, atherogenesis, and CVD development. MGO and MAGE accumulation is observed when the key system responsible for MGO detoxification (Glo1/Glo2) is overloaded. Then MGO reacts with arginine or lysine residues of peptides, proteins, and lipoproteins, yielding stable adducts (e.g., MG-Hs, CEA, AP, THP, CEL, and MOLD). Additionally, it causes the formation of macromolecule cross-links with the involvement of arginine and lysine (MODIC). Moreover, MGO participates in DNA and RNA modification, reacting with deoxyguanosine and inducing nucleic acid cross-linking. Hence, MGO can lead to epigenetic changes through the alteration of genetic material (nucleic acids and/or histones) and induce metabolic memory comprising the prolonged upregulation of prooxidative (ROS increase) and proinflammatory (AGE/RAGE and NF-κB-mediated) pathways. To date, several groups of small molecules have been evaluated for possible MGO-trapping effects, inhibiting glycation or (M)AGE/RAGE signaling through various mechanisms. The glycation inhibitors exhibit the ability to activate the Glo1/Glo2 system, capture MGO and RONS, chelate transition metals that catalyze oxidation, and inhibit the MAGE-RAGE axis by inducing circulating RAGE (sRAGE and esRAGE) and also by other mechanisms (see Figure 4).

### 4.1. Overview of the Potential Glycation Inhibitors and MGO Scavengers

Glycemic control and the inhibition of nonenzymatic glycation are at the heart of the strategies to prevent MGO-induced vascular complications in diabetes. Methylglyoxal at the micromolar level causes rapid injury to endothelial cells and contributes directly to their inflammation and dysfunction in vitro. Fluctuating increases in MGO levels coupled with hyperglycemia spikes also impair angiogenesis in vivo. For this reason, MGO appears to be a critical precursor of AGEs (MAGEs) and AGE-associated disturbances [5,17,155,178,220]. Its neutralization (removal) is probably the principal way in which a number of antiglycation agents inhibit the formation of AGEs (MAGEs). Aminoguanidine (syn. pimagedine) is the first known MGO-trapping glycation inhibitor that effectively prevents the formation of AGEs and reduces the cross-linking of arterial wall proteins in hyperglycemia. In experimental models of diabetes complications, it was effective in inhibiting disease progression. Namely, aminoguanidine prevented vascular dysfunction, reduced renal basement membrane thickening and albuminuria, as well as normalized endothelial cell proliferation in retinopathy. The clinical effect of aminoguanidine was studied in two randomized placebo-controlled trials on more than 600 patients with T2DM and T1DM accompanied by nephropathy or retinopathy. However, these studies were discontinued prematurely due to several side effects [221,222]. Therefore, an intensive search for new glycation inhibitor candidates has been conducted, including screening among commonly used medications to repurpose them for other applications.

To date, several groups of small molecules have been evaluated for possible MGO-trapping effects, inhibiting glycation or AGE/RAGE (MAGE/RAGE) signaling through various mechanisms, including oral antihyperglycemic agents (biguanides, sulfonylureas, thiazolidinediones) used to treat T2DM and insulin resistance; angiotensin II receptor antagonists (angiotensin II receptor blockers) and angiotensin-converting enzyme inhibitors; calcium antagonists (calcium channel blockers) and arterial smooth muscle agents (hydrazinophthalazine derivatives) used to treat hypertension; hypocholesterolemic agents (statins) and phlebotropic agents (vasodilators, anti-varicose, and capillary stabilizing agents) used to treat peripheral vascular disease (also in patients with diabetes); and anti-inflammatory, analgesic, and antipyretic agents used to treat inflammatory joint disease, cold, flu, and headache (including nonsteroidal anti-inflammatory drugs (NSAIDs)), as well as some vitamins (such as B1, B6, and others).

A list of potential and known glycation inhibitors and MGO scavengers, along with proposed mechanisms of antiglycation action, are presented in Table 2. However, only preliminary data derived from in vitro or animal model studies are available to date for most of these inhibitors. Studies involving healthy volunteers or patients have been conducted for metformin [223], atorvastatin [224], cerivastatin [225], benfotiamine [226], pyridoxamine [227], hesperidin [228], and isoquercitrin [212]. Among the compounds discussed in this section, only biguanides, hydrazinophthalazines, and bioflavonoids show the ability to trap MGO with the formation of adducts having a different chemical structure from the precursor (MGO attaches to the scavenger via covalent bonds) [223,229,230,231]. In turn, Voziyan et al. [232] and Colzani et al. [230] confirmed the ability of pyridoxamine to uptake RCS in a test with GO and MDA. The inhibitor’s ability to capture RCS results in the disposal of MGO and other carbonyls, just as circulating RAGE captures and eliminates AGEs. An MGO-metformin metabolite (an imidazolinone derivative) is excreted in the urine of metformin-treated patients [223]; nevertheless, the ultimate fate of the adducts of other scavengers remains unknown.

Because the agents in the discussion are commonly used in primary therapeutic ranges due to their known mechanisms of action, the possibility of their broader use in pharmacotherapy due to the inhibition of the MAGE-RAGE axis is considered. This approach may reduce or slow down the effect of MAGE-RAGE on the development or progression of CVD.

#### 4.1.1. Oral Antihyperglycemic Agents

Blood glucose-lowering agents are known to reduce vascular dysfunction in preclinical models through a combination of mechanisms that apparently act independently of the glucose-lowering benefits. In the group of antihyperglycemic medicines, the effect of lowering AGEs and cross-linking macromolecules by directly reducing MGO levels has been proven only for biguanides (metformin, buformin) [233,234,235,236]. Metformin treatment in patients with T2DM and atherosclerosis, besides its hypoglycemic effect, is associated with increased Glo1 activity. However, there was no effect on Glo1 protein levels, but Glo1 activity correlated with HbA1c levels [236]. Human studies have also shown that metformin can trap MGO (Table 2). The reaction produces an imidazolinone-like metabolite via nucleophilic addition involving a biguanide group [223]. The latter mechanism presumably contributes to a significant reduction in MGO levels and appears to offer therapeutic value in inhibiting MAGE-RAGE (and also AMPK activation).

Sulfonylureas (glibenclamide = glyburide, gliclazide, glipizide, glimepiride) restore cellular antioxidant levels and reduce oxidative stress, resulting in the inhibition of AGE production; however, the antiglycation mechanism for them is not fully known. In the in vitro models (HSA or BSA-glucose, HSA or BSA-MGO), glibenclamide, gliclazide, and glipizide directly inhibited the formation of AGEs (i.e., CML and argpyrimidine), and the strength of this effect was comparable to aminoguanidine [235,238,239]. In the presence of glibenclamide [239], the modification of human plasma albumin by glucose and MGO decreased by 70% (early and advanced glycation products), and the possible mechanism was attributed to interaction with proteins (mechanism (5), protection of glycation sites). Nevertheless, the antiglycation effect of sulfonylurea derivatives in vivo is probably related to their principal direction of action. The hypoglycemic effect of sulfonylurea derivatives results from binding to the sulfonylurea receptor (SUR) and closing ATP-sensitive potassium channels (K_ATP_ channel—K_ATP_, potassium channels activated by a decrease in intracellular ATP and an increase in ADP) in pancreatic β-cells. The depolarization of the cell membrane occurs by impairing potassium excretion and increasing intracellular K^+^, followed by the opening of voltage-gated Ca^2+^ channels and increasing Ca^2+^ influx, which induces insulin secretion [283]. SUR subunits act as sensors of cellular metabolism. K_ATP_ has been identified in the membranes of numerous cell types, including endothelium, cardiomyocytes, and subcellular membranes (mitochondrial, nuclear, sarcolemmal). After an energy crisis in a cell, mitochondrial activity tends to deteriorate. In such a situation, mitochondrial K_ATP_ channels open and close, leading to unbalanced transmembrane ion transport and the overproduction of ROS. In a human aortic endothelial cell (HAEC) line, MGO caused the sustained abnormal activation of K_ATP_ channels and increased their conductance. In addition, MGO exposure potentiated three mitogen-activated protein kinase (MAPK) pathways in HAECs, and glibenclamide reversed the activation of JNK (stress-activated protein kinase) by blocking K_ATP_ (K_ATP_ antagonist). Therefore, it seems that through this mechanism, K_ATP_ blockers may prevent MGO-induced endothelial (and other) cell dysfunction [155]. A depletion of ATP stores and an elevation in AMP levels are also associated with the activation of AMPK (adenosine 5′-monophosphate-activated protein kinase), which plays a pivotal role in the cell’s energy metabolism. Once activated, AMPK inhibits ATP consumption pathways and turns on catabolic ATP production pathways, and through downregulatory signaling pathways and target molecules, modulates carbohydrate and lipid metabolism (glucose uptake, gluconeogenesis, fatty acid oxidation, cholesterol synthesis, lipid synthesis, etc.). In addition, AMPK is involved in the alleviation of oxidative stress, the regulation of autophagy, and the countering of apoptosis. In a study by Lee, Kim, and Choi [240], gliclazide increased the level of phosphorylated AMPK in vascular smooth muscle cells (VSMCs) and inhibited platelet-derived growth factor (PDGF)-induced VSMC proliferation by increasing intracellular Ca^2+^ concentration, which is beneficial for CVD risk reduction. Gliclazide also increased the level of Ca^2+^/calmodulin-dependent protein kinase β (CaMKKβ), an upstream kinase of AMPK.

These results suggest that the effect of K_ATP_ channels on AMPK activity was achieved through the regulation of intracellular Ca^2+^ levels. Oral administration of gliclazide induced activation of CaMKKβ and AMPK in vivo, demonstrating that gliclazide suppressed VSMC proliferation through the CaMKKβ-AMPK signaling pathway. Elucidating the physiological functions of K_ATP_ and AMPK in the cardiovascular system remains an active topic of study [284]. On the other hand, in a randomized controlled trial (PioRAGE), in patients with T2DM, glimepiride increased plasma esRAGE and decreased RAGE expression in peripheral mononuclear cells, but to a lesser extent than pioglitazone [241].

Thiazolidinediones (pioglitazone, rosiglitazone) inhibited the AGE/RAGE pathway and NF-κB as well as alleviated cellular oxidative stress in an in vitro model on isolated rat platelets, human umbilical vein endothelial cells, and human embryonic kidney cells. These effects were associated with a downregulation of RAGE and RAGE mRNA expression and a restoration of cellular antioxidants [242,243,244]. Low sRAGE levels are associated with the incidence of vascular complications in patients with T2DM [285]. Preliminary evidence suggests that some thiazolidinediones may also modulate soluble RAGE levels in a hyperglycemic environment. A study by Oz Gul et al. [245] showed that pioglitazone (but not rosiglitazone) in T2DM patients significantly increased circulating sRAGE levels, which may contribute to its antiatherosclerotic effect. Similar results were obtained by Koyama et al. [241]. On the other hand, Liu and co-authors [243] confirmed that pioglitazone and rosiglitazone inhibit platelet aggregation via AMPK activation. Pioglitazone also preferentially binds to proteins and attenuates structural changes, thereby maintaining their integrity [244].

The potential to prevent hyperglycemia-induced RCS and ROS (or RONS) accumulation can presumably be attributed to other oral blood glucose-lowering agents, including sodium-glucose cotransporter 2 (SGLT2) inhibitors [286].

#### 4.1.2. Angiotensin II Receptor Antagonists, and Angiotensin-Converting Enzyme Inhibitors

Angiotensin II receptor antagonists (candesartan, irbesartan, losartan, olmesartan, telmisartan, valsartan) and angiotensin-converting enzyme inhibitors (captopril, enalaprilat, perindoprilat, temocaprilat) reduce the formation of AGEs by several mechanisms (Table 2), including the activation of the glyoxalase system, antioxidant activity, and the chelation of transition metal cations, which leads to a decrease in the levels of RONS, MGO, and MAGEs [207,208,246,247,248]. The anti-AGE effect of olmesartan and temocaprilat (the active metabolite of temocapril) is not mediated by RCS trapping [247]. Studies by Miller et al. [207,208] demonstrated that that angiotensin II is a negative regulator of Glo1 activity and expression in retinal vascular cells. In bovine retinal endothelial cells, bovine retinal pericytes, and an animal model, candesartan attenuated vascular damage in diabetic retinopathy by restoring Glo1 activity and mRNA for Glo1 to at least control levels. In contrast, it reduced mRNA for TNF-α and iNOS, cellular ^●^NO (nitric oxide) levels below controls and mRNA for both ICAM-1 (intercellular adhesion molecule 1, which is an endothelium- and leukocyte-associated transmembrane protein and is involved in stabilizing cell–cell interactions and facilitating leukocyte transendothelial migration) and VEGF (vascular endothelial growth factor, which is involved in vasculogenesis and angiogenesis) to nondiabetic control levels. The anti-AGE efficacy of candesartan has been confirmed in Ren-2 rats. As a result, the authors observed a reduction in retinal argpyrimidine levels and total AGEs in plasma compared to the control group [208].

#### 4.1.3. Calcium Channel Blockers

Calcium channel blockers (amlodipine, lacidipine, nifedipine, diltiazem, and semotiadil) inhibit glycation and delay AGE formation through mechanisms related to preventing macromolecule oxidation. They generally act as antioxidants and protect, for example, lipoproteins from further modification, but the effect on Amadori product generation is weak. Sobal, Menzel, and Sinzinger [249] compared the antioxidant efficacy of calcium antagonists in preventing the copper-catalyzed oxidation of non-glycated and glycated LDL. The authors showed the significant antioxidant activity of calcium channel blockers during long-term LDL glycation. The oxidation of native LDL was inhibited most efficiently by lacidipine and semotiadil, but only lacidipine significantly inhibited the oxidation of glycated LDL.

#### 4.1.4. Hydrazinophthalazine Derivatives

Hydralazine is an antihypertensive agent (arterial smooth muscle agents) from hydrazinophthalazine derivatives used in the treatment of essential hypertension or severe hypertension associated with conditions requiring immediate action, e.g., heart failure. The mechanism of hydralazine anti-AGE activity was documented by Nangaku et al. [248] and Colzani et al. [230]. Unlike olmesartan, but similar to aminoguanidine, hydralazine effectively captures RCS (i.e., MGO and GO) in vitro. It also impairs oxidative metabolism. Hydralazine reduces the level of carbon-centered radicals in a dose-dependent manner and the concentration of hydroxyl radicals. Finally, hydralazine interrupts the Fenton reaction because it chelates copper and inhibits the autoxidation of ascorbic acid. Inhibiting LDL glycation by trapping reactive carbonyls that induce LDL modification prevents lipid loading and foam cell formation in macrophage cells. RCS scavengers, such as biguanides and hydrazinophthalazines, can inhibit LDL glycation and prevent diabetes-induced atherosclerosis at concentrations equivalent to or above the glycating agent [250].

#### 4.1.5. Lipid Modifying Agents (Statins)

Statins (atorvastatin, cerivastatin, fluvastatin, pitavastatin, pravastatin, rosuvastatin, and simvastatin) are used to lower cholesterol levels by inhibiting hydroxymethylglutaryl-CoA (HMG-CoA) reductase, which is involved in hepatic cholesterol synthesis. The beneficial effects of statin therapy on reducing the pathogenesis of the cardiovascular system, arteriosclerosis, and diabetic complications are commonly known. Studies in animal models have shown that RAGE is the best-known target for AGEs in the cardiovascular system, and the AGE/RAGE pathway contributes to the progression of atherosclerosis [218]. Statins can lower serum AGE levels in a manner that is independent of, but also related to, the hypocholesterolemic effect. The anti-AGE effect of statins may be linked to increased sRAGE levels, decreased RAGE expression (6), and a slight increase in PPAR-γ receptor expression, leading to reduced ROS production and neutrophil adhesion in vitro. Indeed, data obtained in a group of T2DM patients treated with simvastatin showed a significant reduction in ROS production and neutrophil adhesion [213]. Statins appear to activate PPAR-γ by stimulating cyclooxygenase-2 and modulating the Wnt signaling pathway (Wnt proteins are secreted glycoproteins that regulate diverse developmental processes) by inhibiting Dickkopf-related protein 1 (DKK-1), which acts antagonistically to Wnt. PPAR-γ is involved in the modulation of gene transcription and has protective effects on the endothelium by inhibiting endothelin-1 release and mitigating/preventing the inflammatory response [254]. Blocking the AGE/RAGE pathway was also confirmed for pravastatin and rosuvastatin in a model of diabetic nephropathy [256]. Quade-Lyssy et al. [218] connected the anti-AGE activity of statins at low concentrations to their cholesterol-lowering effects. Lovastatin in mouse alveolar epithelial cells endogenously expressing RAGE and human embryonic kidney cells overexpressing RAGE induced sRAGE secretion but did not affect esRAGE secretion. The secretion of sRAGE was also evident after the restoration of the isoprenylation pathway, confirming the correlation between sterol biosynthesis and the activation of RAGE excretion. In contrast, the lovastatin-stimulated RAGE secretion was completely abrogated by the metalloproteinase inhibitor [218]. Similarly, in another model, atorvastatin produced an anti-AGE effect in diabetic nephropathy by increasing the sRAGE level [252]. Furthermore, statins can reduce the level of AGEs by increasing the expression of NAD(P)H dehydrogenase (quinone) 1 (NQO-1) and heme oxygenase 1 (HO-1) genes in the ERK5-dependent Nrf2 (nuclear factor erythroid 2-related factor 2 dependent on extracellular signal-regulated kinase 5) signaling pathway [253,255]. The Nrf2 transcription factor regulates the expression of antioxidant proteins [120], and ERK5/NRF2 signaling plays a significant role in vascular protection against oxidative stress and the maintenance of endothelial integrity [287].

Some metabolites of statins have extra anti-AGE effects. The hydroxyl metabolites of atorvastatin acquire unusual antioxidant properties [251]. They prevent lipoprotein oxidation, and their effect on HDL and LDL is related to the protection of paraoxonase activity (paraoxonase is an enzyme associated with high-density lipoproteins, capable of hydrolyzing lipid peroxides). An accessible multicenter, double-blind, randomized clinical trial of cerivastatin in patients with T2DM confirmed significantly lowered endogenous AGEs (by 21% after 12 weeks, CML was determined using ELISA assay) correlated with reduced LDL cholesterol and apolipoprotein B levels in all LDL subfractions, without effect on HbA1c [225]. After 12 weeks of treatment with cerivastatin, the concentration of oxidized LDL was lowered by 23%. The mechanisms outlined above may underline the anti-inflammatory and anticoagulant effects of statins related to AGEs. They also reinforce the thesis that their use in diabetic angiopathy is warranted to improve endothelial homeostasis [257]. However, further studies are needed to clarify whether the reduction of serum AGEs by statins and their metabolites can reduce the risk of future cardiovascular events. The effect of statins on AGEs was summarized by Niedzielski et al. [288].

#### 4.1.6. Peripheral Vasodilators and Vasoprotectives

For phlebotropic and angioprotective agents (pentoxifylline, calcium dobesylate, and bioflavonoids), mechanisms (1) to (5) have been reported [228,231,237]. Pentoxifylline is a competitive, nonselective inhibitor of phosphodiesterases that increases intracellular cAMP concentrations and possesses anti-inflammatory and antioxidative properties. It exhibits potential for slowing the progression of atherosclerosis, stabilizing plaque, reducing risk, and improving the outcome of vascular events, providing benefits in intermittent claudication and angina, enhancing cerebral blood flow in patients with cerebrovascular disease, improving prognosis in congestive heart failure, and aiding diabetes control. The effect of pentoxifylline on vascular health is summarized by McCarty et al. [289]. In the BSA–glucose model, pentoxifylline, like metformin and pioglitazone, showed a moderate inhibitory effect on the early glycation stage and the ability to inhibit AGE-cross-linking [237].

Calcium dobesylate is a well-known vasoactive agent (in use for more than 40 years) that exhibits a multidirectional mode of action confirmed in laboratory studies, animal models, and clinical trials [259,290]. Several studies have shown the positive effects of dobesylate on endothelial dysfunction, microinflammation, vasoconstriction, and increased vascular permeability. It likely reduces endothelial damage by acting through multiple pathogenic pathways involved in the progression of angiopathy. Nevertheless, the mechanism responsible for this effect has not been definitively elucidated. At the cellular level, dobesylate reduces oxidative stress and inflammation. It can also protect the endoplasmic reticulum and mitochondria and reduce excessive calcium loading, as reflected in a decrease in endothelial cell damage [260]. Deng and colleagues [258] also confirmed that dobesylate reduced endoplasmic reticulum calcium impairment in cultured rat cardiomyocytes at both high glucose and lipid levels. Its anti-glycation and anti-AGEs potential (higher than metformin) was confirmed in vitro in a model with bovine serum albumin and MGO. Although dobesylate does not capture RCS, it protects the albumin protein from modification under carbonyl stress conditions through direct antioxidant activity [231]. On the other hand, the reduction of angiogenesis and capillary permeability, crucial in microvascular disease, is associated with the inhibition of vascular endothelial growth factor (VEGF) and fibroblast growth factor (FGF). Dobesylate recognizes both growth factors, changes their three-dimensional structure at the site of recognition by the receptors (VEGFR and FGFR) and is, therefore, capable of dissociating the receptor–growth factor signaling complex [291]. Finally, the systematic reviews and meta-analyses have confirmed that dobesylate therapy is significantly associated with reduced symptoms of diabetic retinopathy and nephropathy, both at the overall and local levels [261,290,291].

Plant flavonoids (also known as bioflavonoids, included in a broad class of polyphenols with antioxidant properties) like rutin, isoquercitrin, quercetin, hesperidin, hesperetin, diosmin, diosmetin, and their semisynthetic derivatives, e.g., troxerutin, have shown the ability to scavenge ROS and RCS—mainly MGO—in experimental glycation models [229,231]. Generally, compounds in this chemical group capture one or two molecules of MGO and reduce the accumulation of MAGEs. However, some 7-*O*-substituted derivatives, such as diosmin and troxerutin, did not scavenge MGO in vitro but protected the model protein from modifications and AGE formation through antioxidant action. Interestingly, quercetin adducts with MGO retain antioxidant activity and scavenge radicals in a dose-dependent manner [263]. The results of Bhuiyan et al. [229] further suggest that the ability of flavonoids to chelate transition metal cations is significant for their overall anti-AGE activity. Flavonoids are also known to interact with macromolecules (e.g., quercetin forms complexes with albumin protein involving hydrogen bonds and hydrophobic interactions [264]). The formation of nonpersistent complexes in the area of glycation sites can protect the structure of the macromolecule from modification. Besides biguanides and hydrazinophthalazines, bioflavonoids are practically the only glycation inhibitors with the ability to trap MGO (and other RCS) and the formation of stable adducts [229]. To date, results from two placebo-controlled clinical trials have been published that confirmed the ability of flavonoids to lower plasma MGO levels (changes in other RCS were not statistically significant). In a study by Van den Eynde et al. [212] involving subjects with (pre)hypertension, isoquercitrin (quercetin-3-*O*-glucoside 160 mg/day) decreased MGO levels significantly by about 11%; however, there was no significant change in Glo1 expression. A similar result (about a 10% reduction in MGO) was obtained for hesperidin (450 mg/day) in a study by Bednarska et al. [228]. Although the biochemical effect of MGO-trapping flavonoids typically does not exceed 12%, long-term observational studies suggest that even such a small reduction in blood methylglyoxal concentrations can be clinically significant [178,228]. Nevertheless, further work is needed to determine the exact pharmacological effects and optimize dosage.

The positive effects of flavonoids on cardiometabolic health are further confirmed by epidemiological studies. Wang et al. [292], in a systematic review and meta-analysis of 14 prospective cohort studies, found that the intake of flavonoids from different subgroups (flavonols, flavones, flavanones, flavan-3-ols, and others) is inversely associated with cardiovascular disease risk. Liu et al. [263], Kim and Je [265], Grosso et al. [266], and Micek et al. [267] conducted meta-analyses of prospective cohort studies evaluating the effect of flavonoids on the risk of mortality from any cause and cardiovascular disease in the general population. In a study by Liu et al. [263], flavonoids significantly reduced the risk of all-cause mortality by 18% in all adult subjects. A dose-response analysis showed that those ingesting 200 mg/day of total flavonoids had the lowest risk of all-cause mortality. The same authors found a marginally significant association between flavonoid intake and the risk of death from CVD and coronary heart disease (but the studies evaluating the effects of CVD and CHD deaths were in limited numbers). Previous meta-analyses have indicated that a high dietary intake of flavonols (a subgroup of flavonoids, including quercetin derivatives) can reduce the risk of coronary heart disease mortality by 20% [268] and total flavonoids by 15% [269]. According to Kim and Je [265], those with the highest intake of flavonoids had a 14% lower risk of cardiovascular and all-cause death compared to those with the least, while Grosso et al. [266] showed a 26% lower risk from any cause, and increasing the amount of flavonoids by 100 mg/day led to a linear risk reduction of 6% and 4% for overall mortality and CVD, respectively. Micek and colleagues [267] included 39 prospective cohort studies involving 1,501,645 people and a total of 33,637 cases of cardiovascular disease. In the last meta-analysis, the increase in total flavonoid intake was also linearly associated with lower cardiovascular disease risk. However, among the flavonoid subgroups, a higher intake of flavonols and flavones was inversely associated with coronary heart disease risk.

#### 4.1.7. Anti-Inflammatory, Analgesic, and Antipyretic Agents

Nonsteroidal anti-inflammatory agents (acetylsalicylic acid, diclofenac, and ibuprofen) and paracetamol in older preclinical and human studies prevented the modification of lens proteins by carbonylation and nonenzymatic glycation [270,271,272,273]. The protection provided by diclofenac is based on the noncovalent interaction of the medication with serum albumin. There is evidence that diclofenac specifically blocks at least one of the principal glycation sites of human serum albumin [270]. However, these results need to be verified by modern methods. On the other hand, Indurthi, Leclerc, and Vetter [293] suggest that glycation in hyperglycemic patients can significantly alter the pharmacokinetics of diclofenac with possible negative implications for patients. Furthermore, Rasheed et al. [274] demonstrated the anti-AGEs, antioxidant, and transition metal cation chelating potential of oxicams (meloxicam, piroxicam), nimesulide, and mefenamic acid in in vitro tests with glucose and MGO as glycation agents.

#### 4.1.8. Selected B Vitamins

Small clinical trials have shown that B vitamins (thiamine, benfotiamine, pyridoxamine) can help delay the progression of end-stage renal failure due to diabetic kidney disease by inhibiting vascular inflammation and endothelial cell damage. However, a 2015 Cochrane systematic review of clinical trials does not support this evidence. Cochrane experts also do not recommend B vitamins or combinations of B vitamins to delay the progression of end-stage renal disease. One study found thiamine to be beneficial for reducing albuminuria; however, there was no improvement in renal function or blood pressure after B vitamin preparations [294].

Benfotiamine (S-benzoyl thiamine *O*-monophosphate, a lipid-soluble vitamin B1) is a known NADPH oxidase inhibitor that prevents tissue damage in many experimental models. It has been confirmed that benfotiamine not only directly inhibits NADPH oxidase activity but also prevents/inactivates the protein kinase C (PKC) pathway, thereby blocking NF-κB activation in diabetic patients. In addition, the inhibitory effect of benfotiamine on NADPH oxidase may occur indirectly through the activation of transketolase, which finally inhibits NADPH oxidase production and activates antioxidant defense mechanisms [276]. In mice with streptozotocin-induced diabetes, benfotiamine significantly reduced the elevation of MGO, AGEs (MAGEs), RAGE, and collagen cross-linking without affecting hypertriglyceridemia and hypercholesterolemia [277]. A clinical trial in T2DM patients without complications confirmed that it significantly reduces CML-AGEs and maintains normal sRAGE levels (sRAGE was decreased in the placebo group) [226].

The activity of pyridoxamine (vitamin B6) against RCS and AGEs has been confirmed in experimental models, such as BSA-glucose, ribonuclease A-glucose, human hemoglobin-glucose, and in the GO uptake assay [232,275,278,281]. In a study on obese mice, pyridoxamine improved glucose tolerance, insulin metabolism, and vascular dysfunction. Furthermore, it reduced fasting insulin levels and improved insulin sensitivity in obese mice and mice with type 2 diabetes [279,280,282]. Its effects on glycation and metabolic and vascular risk parameters in humans were evaluated in a randomized, placebo-controlled, double-blind study involving subjects with abdominal obesity [227]. Daily doses of 25 and 200 mg of pyridoxamine were metabolically active. The higher dose reduced levels of MGO (9% reduction), AGEs (mainly MAGEs, like MG-H1), and soluble intercellular adhesion molecule-1 (sICAM-1). Both doses decreased the levels of endothelial dysfunction marker and soluble vascular cell adhesion molecule-1 (sVCAM-1) but had no effect on insulin sensitivity or vascular function. The molecular mechanism of the above properties is likely related to MGO uptake (without impact on GO and 3-DG) and the inhibition of MAGE formation. The reduction of adhesion markers seems particularly promising because they are involved in the pathogenesis of endothelial damage and atherosclerosis. A summary of scientific data for B vitamins is presented in [295,296].

## 5. Conclusions and Remarks for Future Research

Cardiovascular diseases (CVD) are presently the main cause of death in the world, and type 2 diabetes is one of the most frequently recognized chronic illnesses. Therefore, it severely deteriorates the population’s health and overloads the health care system. Typical cardiovascular complications in T2DM patients comprise coronary heart disease, ischemic stroke, peripheral artery disease, and heart failure. Therefore, there is an urgent need to elucidate mechanisms for lowering the risk of cardiometabolic disorders, which would allow for the design for efficient methods of prophylaxis and therapy.

Methylglyoxal (MGO) is generated in the human organism mainly as a result of the spontaneous fragmentation of two unstable intermediates of glycolysis and fructolysis: glyceraldehyde 3-phosphate (G3P) and dihydroxyacetone phosphate (DHAP). Additionally, minor quantities of MGO can be derived from the metabolism of acetone, aminoacetone, and threonine, as well as from highly processed foods characterized by high contents of glucose and fructose. An excessive consumption of fructose–glucose syrup has been associated with the development of insulin resistance, hyperglycemia (enhanced by gluconeogenesis), and dyslipidemia (enhanced by de novo lipogenesis resulting in increased level of free fatty acids, as well as hypercholesterolemia and hypertriglyceridemia). These metabolic disturbances lead to disorders such as nonalcoholic fatty liver disease, obesity, metabolic syndrome, and type 2 diabetes. Hyperglycemia observed in diabetics enhances the metabolism of glucose accelerating the generation of MGO and its advanced glycation end products—MAGEs. Moreover, stimulated aerobic glycolysis increases reactive oxygen species (ROS) production, which leads to the inhibition of glyceraldehyde 3-phosphate dehydrogenase (GAPDH). Finally, because glycolysis is inhibited at the trioses level, more G3P and DHAP, and consequently MGO, is produced. Thus, considering MGO-caused damage to blood vessels, T2DM patients show increased risk for cardiovascular complications (angiopathy and cardiomyopathy), which might result from the inefficient control of postprandial glycemia. Only early and intensive glycemia control shows long-term beneficial effects with respect to cardiovascular complications. Increased in diabetes, MGO and (M)AGEs show a deleterious impact on endothelium function, which is associated with the promotion of oxidative stress, low-grade inflammation, atherogenesis, and CVD development. Therefore, the elucidation of mechanisms underlying these pathological processes would allow for the establishment of better prophylactic and therapeutic approaches to deal with these diseases.

MGO and MAGE accumulation is observed when the main system responsible for MGO detoxification (Glo1/Glo2) is overloaded. Then MGO reacts with arginine or lysine, yielding stable hydroimidazolone adducts (MG-H1, MG-H2, MG-H3), CEA, AP, THP, CEL, and MOLD. Additionally, it causes the formation of cross-links with the involvement of arginine and lysine (MODIC). Moreover, MGO participates in the modification of DNA and RNA, reacting with deoxyguanosine and yielding, e.g., CEdG, as well as inducing nucleic acids cross-linking. Hence, MGO can lead to epigenetic changes through the alteration of genetic material (nucleic acids and/or histones) and induce metabolic memory comprising prolonged upregulation of prooxidative (ROS increase) and proinflammatory (NF-κB-mediated) pathways. Also, persistent MGO accumulation in the organism would impair the actions of important regulators of metabolic processes, especially proteins containing arginine residues in their functional units. Such a modification of guanidine groups in Arg side chains of AMP-activated kinase (AMPK) (causing its dysfunction) would explain the shift in balance between catabolic and anabolic processes in favor of the latter (observed in cardiometabolic disorders). However, further experiments are required to test the hypothesis that it is MGO that modifies AMPK.

Experiments aimed at the identification of factors preventing glycation and AGE accumulation in tissues, as well as reversing already generated modifications and cross-links of macromolecules, have been conducted since the beginning of the 1990s. However, despite significant development in experimental techniques, only a few compounds with the ability to trap MGO have been reported in the last 30 years. They include some of the biguanides, hydrazinophthalazines, bioflavonoids, and pyridoxamine. A slightly larger group comprises glycation inhibitors (anti-AGEs factors), such as some biguanides, sulfonylureas, thiazolidinediones, angiotensin II receptor antagonists, angiotensin-converting enzyme inhibitors, calcium channel antagonists, hydrazinophthalazines, statins, vasodilators, vasoprotectives, anti-inflammatory and analgesic agents, as well as B vitamins. These medicines are commonly applied in the pharmacotherapy of hyperglycemia, insulin resistance, atherosclerosis, hypertension, peripheral artery disorder, and inflammatory conditions. They are applied as first-choice medicines (blood glucose-lowering agents, or agents for the treatment of cardiovascular conditions) or complementary therapeutics for cardiometabolic conditions (e.g., bioflavonoides, calcium dobesylate, pirydoxamine, benfotiamine). However, studies on factors capable of the degradation of cross-links in modified macromolecules are in their infancy. Therefore, considering their potential in reversing MGO-caused damage in biological components, such experiments should be intensified to yield new therapeutics that will help in MAGE scavenging.

The main mechanisms involved in antiglycation and MGO trapping actions presented in this review are meant to chart a path in search of new molecules characterized with high therapeutic potential. Furthermore, considering the available scientific data, a well-designed clinical trial should be performed to confirm the anti-MGO/MAGEs effects of reported glycation inhibitors. Finally, it would be interesting to learn whether MGO-capture agents, such as metformin, hydralazine, pyridoxamine, quercetin, and hesperidin, can compete with Glo1 in the same compartments and how the kinetics of the trapping reaction affects the binding efficiency of methylglyoxal.

## Figures and Tables

**Table 1:** Methylglyoxal and its AGEs in cardiometabolic disorders: data from rodent models.

Experimental Model	MGO/MAGEs and Associated Major Findings	Ref./Year
SD rats treated with 60% Fru (in chow) for 16 weeks	Upon Fru treatment (in comparison with control SD rats): Increase in systolic blood pressure. In blood serum: Increase in MGO; Decrease in GSH. In aorta: Increase in MGO, hydrogen peroxide and CEL; Decrease in eNOS in endothelial cells; No change in GSH. In mesenteric artery: Increase in CEL and CML; Increase in wall thickness and decrease in the vessel lumen.	[31]/2008
Lean, obese, and diabetic Zucker rats	In obese and/or diabetic Zucker rats (as compared to lean Zucker rats control): In the serum: Increase in Glc, MGO, and fructose; Increase in insulin in obese rats, but decrease in insulin in diabetic rats. In aortas: Increase in MGO and fructose; Upregulation of GLUT-5, fructokinase, and aldolase B (at mRNA levels); Increase in aldose reductase and sorbitol in diabetic rats.	[89]/2011
Sprague Dawley (SD) rats treated with 60% fructose (Fru) (in chow) for 16 weeks	Upon Fru treatment (in comparison with control SD rats): In aortas: Increase in MGO and Fru; Upregulation of GLUT-5, fructokinase, and aldolase B (at mRNA levels).	[89]/2011
SD rats treated with Fru-enriched diet (60% fructose, 22% crude proteins, 5% crude fat, 5% crude fiber, 6% ash, and 2% minerals) for 9 weeks	Upon Fru treatment (in comparison with control SD rats): Increase in blood pressure. In blood serum: Increase in MGO, TAG, and insulin; No changes in total cholesterol, HDL-cholesterol, HbA1c, or Glc. In the adipose tissue: Increase in MGO and PI3K protein; Decrease in insulin-induced Glc uptake; Decreased PI3K recruitment to phosphorylated IRS-1; No changes in IR, IRS-1 expression, or phosphorylation.	[94]/2007
SD rats treated with 60% Fru (in chow) for 16 weeks	Upon Fru treatment (in comparison with control SD rats): Increase in blood pressure and vascular remodeling; In blood plasma: Increase in MGO. In aorta: Increase in MGO and Akt1 phosphorylation at Ser-473.	[95]/2011
SD rats treated with MGO (administered using continuous infusion with a minipump for 4 weeks; 60 mg/kg/day)	Upon MGO treatment (in comparison with control rats): In aorta: Increase in Akt1 phosphorylation at Ser-473.	[95]/2011
Wistar rats infused with MGO (75 mg/kg body weight/day for 8 weeks)	Upon MGO treatment (in comparison with control rats): In blood plasma: increase in CML. In heart tissue: Decrease in catalase, SOD, and GSH; Increase in cardiac fibrosis; Upregulated expression of RAGE (3.5 fold), TGF-β (4.4 fold), SMAD2 (3.7 fold), SMAD3 (6.0 fold), IL-6 (4.3 fold), and TNF-α (5.5 fold).	[96]/2017
Wistar rats fed with MGO diluted in the daily water (75 mg/kg/day) for 8 weeks	Upon MGO treatment (in comparison with control rats): In blood plasma/serum: Increase in free fatty acids; No change in glycemia (fasting and 2 h after glucose administration), glycated haemoglobin, insulinemia and total cholesterol, triglycerides, and adiponectin levels. In the adipose tissue: Increase in CEL and fibrosis. In the adipose tissue of MGO-fed Wistar rats after blood supply reduction: Increase in ERK1/2 phosphorylation (p-ERK1 plus p-ERK2); Increase in perilipin A degradation (due to MGO-induced glycation); Decrease in IkBa, PPARγ expression, and Akt activation.	[97]/2013
Wistar rats treated with MGO (administered intraperitoneally over 5 consecutive days each week for 7 weeks: 50 mg/kg for weeks 1 and 2; 60 mg/kg for weeks 3 and 4; 75 mg/kg for weeks 5, 6 and 7)	Upon MGO treatment (in comparison with control rats): In the blood serum: Increase in cholesterol, creatinine, and fructosamine; No change in Glc; In skin vasculature: Upregulation of AGEs and RAGE; Progressive thickening of blood vessel wall followed by its detachment from matrix, luminal occlusion, and endothelial cell death ending up with vessel disappearance; No vasodilation upon nitroglycerine treatment; Proinflammatory and profibrotic response (increased IL-1β, TNF-α, CTGF, and TGF-β; disturbances in wound healing).	[98]/2005
SD rats treated with MGO (1% MGO in tap drinking water) for 4 weeks	Upon MGO treatment (in comparison with control rats): Increase in insulin resistance without increase in blood pressure. In the kidney: Increase in CEL and nitrotyrosine.	[99]/2009
SD rats treated with MGO (17.25 mg/kg in a single intraperitoneal injection, or 6.48/50 mg/kg as intravenous infusion)	Upon MGO treatment (in comparison with control rats): Impairment in Glc tolerance. In the blood plasma: Increase in insulin; Decrease in glutathione. In the visceral adipose tissue: Decrease in insulin-stimulated glucose uptake; Reduced plasma membrane GLUT-4 and IRS-1 tyrosine phosphorylation; No change in insulin receptor and IRS-1 protein expression.	[100]/2010
C57/BL6 mice treated with MGO (administered intraperitoneally over 5 consecutive days each week for 7 weeks: 50 mg/kg for weeks 1 and 2; 60 mg/kg for weeks 3 and 4; and 75 mg/kg for weeks 5, 6, and 7)	Upon MGO treatment (in comparison with control mice): Increase in systemic insulin resistance. In the blood serum: Reduction of insulin-stimulated increase in serum NO. In aortas: Decrease in insulin-induced activation of Akt and eNOS; Induction of ERK ½ phosphorylation and endothelin-1 release (comparable to insulin effect).	[101]/2014
C57/BL6 mice treated with MGO (administered intraperitoneally over 5 consecutive days each week for 7 weeks: 50 mg/kg for weeks 1 and 2; 60 mg/kg for weeks 3, 4, and 5; and 75 mg/kg for weeks 6 and 7)	Upon MGO treatment (in comparison with control mice): In the blood serum/plasma: Decrease in the levels of SOD, CAT, and GPX; Increase in MDA; Increase in proinflammatory cytokines (IL-1β and IL-6) and the anti-inflammatory cytokine IL-10. In aortas: Increase in aorta thickness and apoptosis; Decrease in Nrf2 expression and Akt phosphorylation.	[102]/2022
SD rats treated with MGO (administered using continuous infusion with a minipump for 4 weeks; 60 mg/kg/day)	Upon constant MGO treatment (in comparison with control rats): In the blood plasma: Increase in Glc, total cholesterol, TAG, and free fatty acids; Decrease in fasting insulin, HDL, and GSH. In the pancreas/pancreatic β-cells: Enhanced formation of CML and increased apoptosis; Reduced GLUT-2 (=decreased Glc uptake) and glucokinase; Lowered insulin secretion—downregulation of factors promoting insulin expression (PDX-1 and MafA) and upregulation of the factor inhibiting insulin expression (C/EBPβ); Upregulation of NF-κB and RAGE. In the adipose tissue: Decrease in insulin-stimulated Glc uptake; Reduced plasma membrane GLUT-4, IRS-1 phosphorylation, and PI3K activity; No change in insulin receptor or IRS-1 protein expression. Decrease in GSH in pancreas and skeletal muscle.	[103]/2011
SD rats treated with MGO (administered using continuous infusion with a minipump for 4 weeks; 24 mg/day)	Upon constant MGO treatment (in comparison with control rats): Increase in blood pressure. In blood plasma: Increase in norepinephrine, epinephrine, dopamine, angiotensin, renin, and aldosterone. In aortas: Elevated adrenergic α_1D_ receptor, angiotensin AT1 receptor, and angiotensin protein and mRNA. In the kidney: Increase in angiotensin AT1 receptor, renin, and angiotensin protein and mRNA. In aortas and kidney: Increase in phosphorylated Erk 1/2 (p-Erk 1/2) and NFATc expression.	[104]/2014
Wistar rats treated with MGO (50–75 mg/kg/day, in drinking water for 3 months)	Upon MGO treatment (in comparison with control Wistar rats): In aorta: Decline in NO-dependent vascular relaxation; Increase in superoxide, nitrotyrosine, MCP-1, AGEs, and RAGE. In urine: Increase in 8-OHdG.	[105]/2012
Goto–Kakizaki (GK) rats (spontaneously diabetic—T2DM) treated with MGO (50–75 mg/kg/day, in drinking water for 3 months)	Upon MGO treatment (in comparison with control GK rats): In aorta: Decline in NO-dependent vascular relaxation; Increase in nitrotyrosine and RAGE. In urine: Increase in 8-OHdG.	[105]/2012
Wistar rats treated with MGO (50–75 mg/kg/day, in drinking water for 14 weeks)	Upon MGO treatment (in comparison with control Wistar rats): In blood plasma/serum: Increase in free fatty acids; Decrease in adiponectin. In urine: Increase in 8-OHdG. In the adipose tissue: Increase in MGO, AGEs, glycoconjugates, fibrosis, and TGF-β (but not its cleaved form); Increase in proapoptotic factors (decreased Bcl2/Bax ratio and upregulation of caspase 3); Increase in proinflammatory factors (MCP-1 and F4/80); Decrease in VEGF but unchanged angiopoietin 2.	[106]/2012
Hereditary hypertriglyceridaemic rats (HHTg) treated with MGO (administered intragastrically three times a week at a dose of 0.5 mg/kg for 4 weeks)	Upon MGO treatment (in comparison with control HHTg rats): In blood serum: Increase in non-fasting Glc and insulin; Increase in proinflammatory MCP-1 and TNFα. In white adipose tissue: Decrease in the conversion of Glc into lipids upon insulin stimulation; Increase in adrenaline-stimulated lipolysis; Increase in saturated fatty acids and decrease in polyunsaturated fatty acids; Decrease in Nrf2 expression; Increase in MCP-1 and TNFα expression; No effect on Glo1 or HIF-1 expression.	[107]/2020
Spontaneously hypertensive rats (SHR) and Wistar Kyoto rats (WKY)	In comparison with normal WKY, in SHR rats: Higher MGO level in blood plasma and kidney (increasing with age); Higher CML and CEL staining in the kidney; Decreased GSH and GSH/GSSG ratio in the kidney of the oldest 20-week rats.	[108]/2004
SHR and WKY rats	In comparison with normal WKY, in SHR rats: Higher MGO level in blood plasma and aorta (increasing with age); Higher MGO level in the liver and kidney (but not in the heart) in 13-week rats; Higher CML and CEL staining in the aorta (mostly in endothelial cells, lower in smooth muscle cells); Increased oxidative stress (superoxide anion and hydrogen peroxide) in 13-week rats aortas; Decreased GSH in 13-week rats’ aortas; Decreased activities of glutathione peroxidase and reductase in 13-week rats’ aortas; Increased activity of SSAO in blood plasma; No difference in blood plasma GSH.	[109]/2005
SHR and WKY rats	In comparison with normal WKY, in SHR rats: Higher MGO level in blood plasma and aorta; Higher level of AP and CEL in the aorta and mesenteric artery; Increase in oxidative stress in aortic tissue (enhanced level of superoxide anion and nitric oxide, decreased GSH); Changes in nitric oxide synthases expression in aortic tissue (increase in iNOS and decrease in eNOS); Worsening of morphologic changes and endothelium-dependent relaxation in mesenteric artery.	[110]/2007
SHR and WKY rats	In comparison with normal WKY, in SHR rats: Higher levels of AP and CEL in the mesenteric artery; Increase in oxidative stress in mesenteric artery.	[111]/2012
SHR and WKY rats	In comparison with normal WKY, in SHR rats: In the serum: Similar Glc, increase in MGO, fructose, and insulin; In aortas: Increase in MGO and fructose; Upregulation of GLUT-5, fructokinase, and aldolase B (at mRNA levels).	[89]/2011

**Table 2:** Potential and known glycation inhibitors and MGO scavengers with their possible mechanism of action; medications have been grouped according to the Anatomical Therapeutic Chemical (ATC) classification system.

Medications (Agents)	Possible Mechanism of Action: Key Points in the MAGE/RAGE or AGE/RAGE Axis, Biochemical and Physiological Effects	Ref.
1. Antihyperglycemic agents used in the treatment of type 2 diabetes (blood glucose-lowering agents)		
Biguanides		
Metformin	In vitro: MGO scavenger, inhibits carbonyl stress; reduces ↓ cross-linking, ↓ AGE, and ↓ HbA1c formation; restores antioxidant levels in monocytes (THP-1 cells) and erythrocytes; reduces mitochondrial complex I activity; activates ↑ AMPK. Human studies: dose-dependently reduces plasma ↓ MGO; increases ↑ Glo1 activity in peripheral blood cells and atherosclerotic lesions; scavenges MGO to form imidazolinone metabolite, which is excreted in urine.	[17] [102] [223] [233] [234] [235] [236] [237]
Buformin	In vitro: reduces ↓ AGE formation (a more potent inhibitor than metformin) and ↓ cross-linking.	[234]
Sulfonylureas		
Glibenclamide (=glyburide)	In vitro *and Animal studies*: reduces ↓ AGE formation; K_ATP_ channel antagonist; reverses the activation of JNK (stress-activated protein kinase) by blocking K_ATP_; reduces endothelial cell dysfunction by inhibiting activation of the JNK/p38 MAPK pathway (study in HAECs from healthy and T2DM donors), and the effect is mediated in part via K_ATP_ and protection of glycation sites.	[155] [238]
Gliclazide	In vitro *and Animal studies*: reduces ↓ AGE formation; K_ATP_ channel antagonist; induces activation of CaMKKβ (Ca^2+^/calmodulin-dependent protein kinase kinase β) and AMPK; inhibits vascular smooth muscle cell proliferation through the CaMKKβ-AMPK pathway; effects of K_ATP_ on AMPK activity are mediated by the regulation of intracellular Ca^2+^ levels.	[239] [240]
Glipizide	In vitro: reduces ↓ AGE formation; restores antioxidant levels in monocytes (THP-1 cells) and erythrocytes.	[235]
Glimepiride	*Human studies*: increases plasma ↑ esRAGE and decreases ↓ RAGE expression in peripheral mononuclear cells (to a lesser extent than pioglitazone).	[241]
Thiazolidinediones		
Pioglitazone	In vitro: reduces ↓ AGE and ↓ HbA1c formation; reduces ↓ RAGE and ↓ RAGE mRNA expression in human endothelial cells, thereby limiting EC susceptibility to proinflammatory AGE effects; suppresses NF-κB levels and alleviates cellular oxidative stress and inflammation; preferentially binds to protein and alleviates protein conformational changes; pioglitazone restores cellular antioxidants and reduces levels of IL-6 and TNF-α by decreasing expression of membrane RAGE and NF-κB; pioglitazone and rosiglitazone inhibit platelet aggregation by activating ↑ AMPK. *Human studies*: increases in circulating ↑ sRAGE or sRAGE/esRAGE; this effect is not observed with rosiglitazone; pioglitazone suppresses RAGE expression and increases circulating sRAGE/esRAGE (activity is not necessarily dependent on plasma glucose or insulin resistance levels).	[241] [242] [243] [244] [245] [237]
Rosiglitazone		
2. Agents for the treatment of cardiovascular conditions		
Angiotensin II receptor antagonists (blockers, ARBs) and angiotensin-converting enzyme inhibitors		
*ARBs* Candesartan Irbesartan Losartan Olmesartan Telmisartan Valsartan	In vitro *and Animal studies*: reduces ↓ AGE (argpyrimidine, pentosidine and CML) formation; chelates transition metal cations, acts as an antioxidant, inhibits ↓ ROS and ↓ RCS formation; the effect on AGE formation is common to all tested ARBs; a similar but milder effect is observed with ACE inhibitors (IC_50_ of pentosidine formation in BSA-arabinose model: valsartan > candesartan > olmesartan > temocaprilat > enalaprilat > irbesartan = losartan = telmisartan > captopril > perindoprilat); Candesartan attenuates vascular dam age in diabetic retinopathy by restoring Glo1 function and reducing ↓ ^●^NO; restores both ↑ Glo1 activity and ↑ Glo1 mRNA levels; reduces ↓ mRNA levels of ICAM-1 (intercellular adhesion molecule), VEGF (vascular endothelial growth factor), TNF-α, and iNOS; reduces ↓ total AGEs, MAGEs, and argpyrimidine in retina and plasma; Olmesartan dose-dependently reduces the development of diabetic nephropathy in rats with type 2 diabetes, as evidenced by reductions in proteinuria and pathologic evidence of diabetic glomerulosclerosis.	[207] [208] [246] [247] [248]
*ACE inhibitors* Captopril Enalaprilat (active metabolite of enalapril) Perindoprilat (active metabolite of perindopril) Temocaprilat (active metabolite of temocapril)		
Calcium channel antagonists (blockers)		
*With vascular effects* Amlodipine Isradipine Lacidipine Nifedipine	In vitro: acts as an antioxidant (lacidipine > semotiadil > amlodipine > nifedipine > diltiazem); inhibits ↓ glycation and ↓ glycoxidation; inhibits the copper-mediated oxidation of non-glycated and glycated LDL.	[249]
*With direct cardiac effects* Diltiazem		
Semotiadil (experimental)		
Hydrazinophthalazine derivatives (arteriolar smooth muscle, agents acting on)		
Hydralazine	In vitro: MGO scavenger; inhibits carbonyl stress; inhibits the formation of AGEs (pentosidine and CML); chelates transition metal cations, acts as an antioxidant, and inhibits the formation of ↓ ROS; inhibits the glycation of LDL and prevents the formation of model foam cells from RCS-modified low-density lipoproteins. *Animal studies*: the effect of hydralazine (5 mg) is similar to that of olmesartan (1 mg), but reached statistical significance only for renal pentosidine content.	[230] [248] [250]
Statins (lipid modifying agents, HMG-CoA reductase inhibitors)		
Atorvastatin	In vitro: atorvastatin *o*- and *p*-OH metabolites are potent antioxidants and protect LDL, VLDL, and HDL from oxidation; the inhibitory effects of these metabolites on HDL oxidation are associated with the protection of paraoxonase activity. *Animal studies*: reduces ↓ AGEs, effect is associated with upregulation of serum and renal ↑ sRAGE levels, although renal esRAGE mRNA expression is not significantly increased. *Human studies*: reduces serum levels of ↓ AGEs in hypercholesterolemic T2DM patients without CVD, but does not reduce fasting glucose or HbA1c levels; AGE changes do not correlate with lipid parameters; atorvastatin tends to reduce serum levels of 8-OHdG (8-hydroxy-2-deoxyguanosine), but not significantly.	[224] [251] [252]
Lovastatin	In vitro: increases the levels of ↑ sRAGE by enhancing the shedding of full-length RAGE, but does not affect the secretion of esRAGE.	[218]
Cerivastatin	*Human studies*: reduces levels of ↓ CML-derived AGEs (compared to the placebo group); effect on CML-AGEs correlates with reduction in LDL cholesterol and LDL apolipoprotein B; HbA1c is not changed.	[225]
Fluvastatin	In vitro: inhibits mitogen-activated protein kinase kinase ↓ MEK (MAPK/ERK kinase, also known as MAP2K, MAPKK), which downregulates EGR-1 (early growth response protein 1) transcription and leads to decreased levels of CTGF (connective tissue growth factor), and consequently reduces proliferation, migration, and ECM (extracellular matrix) accumulation in AGE-induced human aortic smooth muscle cells (VSMCs); activates ↑ PPAR-γ in HASMCs, but not in HUVECs; induces COX-2 expression in HASMCs, but not in HUVECs; suppresses migration and proliferation of HASMCs and inhibits lipopolysaccharide-induced expression of MCP-1 (monocyte chemoattractant protein-1) and TNF-α in HASMCs. *Animal studies*: inhibits atherosclerotic lesion formation in Apoe^−/−^ mice; increases transcriptional activity of ↑ PPAR-γ; and decreases aortic expression of ↓ MCP-1 and ↓ TNF-α.	[253] [254] [255]
Pitavastatin		
Pravastatin	In vitro: inhibits ↓ AGE-induced upregulation of RAGE mRNA levels; reduces ROS generation and apoptosis in human renal proximal tubular cells.	[256]
Rosuvastatin		
Simvastatin	In vitro: reduces ↓ AGE-induced oxidative stress (ROS overproduction) in endothelial cells; diminishes neutrophil adhesion to endothelium; reduces ↓ RAGE mRNA expression, and non-statistically increases ↑ PPAR-γ mRNA expression (PPAR-γ has a protective effect on ECs by inhibiting endothelin-1 release and attenuating/preventing the endothelial inflammatory response). *Animal studies*: 12-week treatment attenuates AGE-induced proliferation of aortic smooth muscle cells in Sprague Dawley rats and reduces ↓ NF-κβ and ↓ MAPK activation in these cells.	[213] [257]
Peripheral vasodilators (purine derivatives)		
Pentoxifylline	In vitro: reduces ↓ AGE and ↓ HbA1c formation	[237]
Vasoprotectives (e.g., for the treatment of peripheral vascular disease)		
*Antivaricose agents* Calcium dobesylate	In vitro: reduces ↓ AGE formation; ROS scavenger; inhibits ↓ ROS formation, acts as an antioxidant; protects glycation reaction substrates from ROS and MGO-induced modifications; reduces impairment of sarcoplasmic reticulum calcium handling and ↓ ROS formation in rat cardiomyocytes caused by high glucose and high lipid levels. *Human studies and Meta-analyses*: reduces blood-retinal barrier permeability as measured by the posterior vitreous penetration ratio (PVPR); effect is manifest regardless of the degree of metabolic control and the use of antihypertensive and lipid-lowering agents; has a significant beneficial effect on the control of hemorrhages and the overall development of diabetic retinopathy.	[231] [258] [259] [260] [261] [262]
*Bioflavonoids (capillary stabilizing agents)* Diosmin (diosmetin-7-O-rutinoside) Hesperidin (hesperetin-7-O-rutinoside) Rutin (quercetin-3-*O*-rutinoside) Troxerutin (trihydroxyethylrutin) Isoquercitrin (quercetin-3-*O*-glucoside)	In vitro: MGO scavenger (not diosmin or troxerutin), ROS scavenger, inhibits the formation of ↓ ROS and ↓ RCS, acts as an antioxidant, chelates transition metal cations; reduces ↓ AGE formation; *Human studies*: quercetin-3-*O*-glucoside and hesperidin reduce plasma ↓ MGO levels (by ~10–11%) in (pre)hypertensive and healthy subjects; but Glo1 expression is not significantly altered; The combination of hesperidin and trans-resveratrol (tRES-HESP) induces ↑ Glo1 expression and counteracts MGO accumulation in overweight and obese subjects; reverses insulin resistance, improves dysglycemia and low-grade inflammation; MGO metabolic variables correlate with BMI, dysglycemia, vascular inflammation, blood pressure, and dyslipidemia. *Epidemiologic studies and Meta-analyses*: the increase in total flavonoid intake is linearly associated with a lower risk of cardiovascular disease; dose-response analysis showed that consumption of 200 mg/day of total flavonoids was associated with the lowest risk of all-cause mortality.	[210] [212] [228] [229] [231] [263] [264] [265] [266] [267] [268] [269]
3. Anti-inflammatory, analgesic and antipyretic agents (including nonsteroidal anti-inflammatory drugs NSAIDs)		
Acetylsalicylic acid (=aspirin)	In vitro: reduces ↓ glycation of albumin and hemoglobin (not salicylic acid), blocks at least one of the major glycation sites of HSA. *Animal studies*: reduces glycohemoglobin and glycoalbumin levels in diabetic rats. *Human studies*: low doses protect against cataracts.	[270] [271] [272]
Diclofenac	In vitro: reduces ↓ glycation of albumin and hemoglobin, blocks at least one of the major glycation sites of HSA.	[270]
Ibuprofen	In vitro: prevents modification of lens proteins by carbonylation and nonenzymatic glycation; reduces cyanate and galactose binding but not glucose-6-phosphate binding; protects against opacities; appears to have a different mechanism of action than aspirin. *Human studies*: low doses protect against cataracts.	[272] [273]
Nimesulide Mefenamic acid Meloxicam Piroxicam	In vitro: reduces ↓ AGE formation; acts as an antioxidant, chelates transition metal cations.	[274]
Paracetamol	*Human studies*: low doses protect against cataracts.	[272]
4. Selected B vitamins		
Thiamine pyrophosphate (B1)	In vitro: reduces ↓ AGE formation (thiamine and thiamine monophosphate are not inhibitors); is essential for maintaining cellular defenses against oxidative stress.	[275]
Benfotiamine (a lipid soluble thiamine derivative)	*Animal studies*: reduces ↓ AGE formation; activates antioxidant defense mechanisms, a ↓ NADPH oxidase inhibitor (this enzyme plays an essential role in ROS production and myocardial cytotoxicity); improves markers of oxidative stress, inflammation, and apoptosis; inhibits ↓ NF-κB by activating transketolase in diabetic animals, prevents experimental diabetic retinopathy; attenuates or abolishes diabetes-induced increase in cardiac levels of ↓ MGO, ↓ AGEs (MAGEs), ↓ RAGE, and ↓ cross-linked collagen without affecting hypertriglyceridemia and hypercholesterolemia. *Human studies*: reduces ↓ CML-derived AGE levels.	[226] [276] [277]
Pyridoxamine, pyridoxal, pyridoxal phosphate, pyridoxine (B6)	In vitro: GO and MDA scavenger, reduces ↓ AGE formation; reduces ↓ ALE formation (but pyridoxine is slightly effective at the highest concentrations). *Animal studies*: inhibits the ↓ AGE/RAGE pathway; increases ↑ Glo1 expression in visceral and perivascular adipose tissue; pyridoxamine reduces MGO-induced atherosclerosis and inflammation; improves glucose tolerance and insulin metabolism in obese mice; prevents adipose tissue inflammation and vascular dysfunction; reduces fasting insulin levels and improves insulin sensitivity in obese and type 2 diabetic mice, most likely by scavenging MGO and inhibiting AGE formation. *Human studies*: reduces ↓ MGO (9%), ↓ MAGEs (MG-H1), ↓ sVCAM-1 (soluble vascular cell adhesion molecule-1), and ↓ sICAM-1 (soluble intercellular adhesion molecule-1), but does not affect insulin sensitivity or vascular function in abdominally obese subjects; reduction of adhesion markers is promising in the pathogenesis of endothelial damage and atherosclerosis.	[209] [220] [227] [230] [232] [275] [278] [279] [280] [281] [282]

AGEs, advanced glycation end products; AMPK, 5′ AMP-activated protein kinase; AOPP, advanced oxidation protein products; BSA, bovine serum albumin; cDNA, complementary DNA; cGMP, cyclic GMP; CML, *N*^ε^-(1-carboxymethyl)lysine; CML-AGEs, AGEs with CML; ELISA, enzyme-linked immunosorbent assay; Glo1, glyoxalase 1; GO, glyoxal; HAECs, human aortic endothelial cells; HbA1c, glycated hemoglobin; HPLC, high-performance liquid chromatography; HUVECs, human umbilical vein endothelial cells; HSA, human serum albumin; IL-6, interleukin-6; KATP, ATP-sensitive potassium channel; LC-MS, liquid chromatography-mass spectrometry; MAPK, mitogen-activated protein kinase; MDA, malonyl dialdehyde; MG-H1, *N*^δ^-(5-hydro-5-methyl-4-imidazolon-2-yl)-ornithine; MGO, methylglyoxal; MS, mass spectrometry; NADPH, reduced form of nicotinamide adenine dinucleotide phosphate; NF-κB, nuclear factor kappa-B; NMR, nuclear magnetic resonance spectroscopy; iNOS, inducible nitric oxide synthase; PCR, polymerase chain reaction; PPAR-γ, peroxisome proliferator-activated receptors-gamma; qPCR, quantitative real-time polymerase chain reaction; RAGE, receptor for advanced glycation end products; sRAGE, soluble RAGE; esRAGE, endogenous secretory RAGE; RCS, reactive carbonyl species; RCT, randomized clinical trial; ROS, reactive oxygen species; RT-PCR, reverse transcription polymerase chain reaction; TNF-α, tumor necrosis factor-α; T2DM, type 2 diabetes mellitus.

## Data Availability

Not applicable.

## Abbreviations

ACh	acetylcholine
AG	aminoguanidine
AGEs	advanced glycation end products
AKRs	Aldoketo reductases
Akt	PKB = protein kinase B (serine/threonine kinase)
ALDHs	Aldehyde dehydrogenases
AMPK	AMP-activated kinase
Ang II (Ang-2)	angiotensin II
AP	argpyrimidine
apoA1	apolipoprotein A1
apoB100	apolipoprotein B100
ApoE KO	apolipoprotein E knockout
ATF6	activating transcription factor 6
AT2R	angiotensin II receptor type 2
BBGC	bromobenzyl-glutathione cyclopentyl diester (glyoxalase-1 inhibitor)
BMCs	bone marrow cells
CAD	coronary artery disease
CAT	catalase
CEA	*N*^7^-carboxyethyl arginine
C/EBP	transcription factor C/EBP
CEdG	*N*^2^-carboxyethyl-20–deoxyguanosine
CEL	*N*^ε^-(1-carboxyethyl)lysine = *N*^6^-(1-carboxyethyl)lysine
*C. elegans*	Caenorhabditis elegans
CETP	cholesteryl ester transfer protein
CKD	chronic kidney disease
CML	*N*^ε^-(1-carboxymethyl)lysine = *N*^6^-(1-carboxymethyl)lysine
CTGF	connective tissue growth factor
CHD	coronary heart diseases
CVD	cardiovascular diseases
DAG	diacylglycerol
DJ-1 (PARK7)	Parkinson’s disease protein 7
DKO	double knockout
3-DG	3-deoxyglucosone
3DG-H	3-DG-derived hydroimidazolones
EA.hy926	hybrid human umbilical vein endothelial cell line
ECM	extracellular matrix
eNOS	endothelial nitric oxide synthase
EPCs	endothelial progenitor cells
ER	endoplasmic reticulum
esRAGE	endogenous secretory RAGE proteolytically exfoliated by metalloproteinases
FFAs	free fatty acids
FL	*N*^ε^-fructosyl-lysine
FPG	fasting plasma glucose
Fru	fructose
F4/80	EGF-like module-containing mucin-like hormone receptor-like 1
GAPDH	glyceraldehyde-3-phosphate dehydrogenase
Glc	glucose
GlcNAc	N-acetylglucosamine
Glo1	Glyoxalase 1
Glo1 KO	Glo1 knockout
Glo2	Glyoxalase 2
GLUT	glucose transporter
GO	glyoxal
GPX	glutathione peroxidase
GSH	reduced glutathione
GSK-3	Glycogen synthase kinase-3
GSSG	oxidized glutathione
HAECs	human aortic endothelial cells
HbA1c	hemoglobin A1c
HEK293	human embryonic kidney cells
HIF	hypoxia-inducible factor
HoxA5	homeobox A5 transcription factor
HO-1	heme oxygenase 1
HSPG	heparan sulfate proteoglycan
HUVECs	human umbilical cord vein endothelial cells
ICAM-1	intercellular adhesion molecule 1
IFN-γ	interferon gamma
IL-6	interleukin 6
IL-8	interleukin 8
IL-1β	interleukin-1 β
IR	insulin receptor
IRE1	inositol-requiring enzyme-1
IRS-1	insulin receptor substrate 1
K_ATP_ channel	ATP-sensitive potassium channel
KRAS	GTPase Kirsten Rat Sarcoma Viral Oncogene Homolog
LCAT	lecithin-cholesterol acyltransferase
Mac-1	macrophage-1 antigen
Mac-2	macrophage-2 antigen
MAECs	mouse aortic endothelial cells
MafA	musculoaponeurotic fibrosarcoma oncogene family A
MAGEs	MGO-derived AGEs
MCP-1	monocyte chemoattractant peptide-1
MDA	malondialdehyde
MG-dG	3-(20–deoxyribosyl)-6,7-dihydro-6,7-dihydroxy-6/7-methylimidazo-[2,3-b]purin-9(8)one
MG-H1-3	MGO-derived hydroimidazolones 1-3
MG-H1	*N^δ^*-(5-hydro-5-methyl-4-imidazolon-2-yl)-ornithine
MG-H2	2-amino-5-(2-amino-5-hydro-5-methyl-4-imidazolon-1-yl)-pentanoic acid
MG-H3	2-amino-5-(2-amino-4-hydro-4-methyl-5-imidazolon-1-yl)-pentanoic acid
MGO	methylglyoxal
MMP-9	matrix metalloproteinase 9
MnSOD	manganese superoxide dismutase
MODIC	2-ammonio-6-((2-[(4-ammonio-5-oxido-5-oxopentyl)amino]-4-methyl-4,5-dihydro-1H-imidazol-5-ylidene)amino)hexanoate
MOLD	1,3-di(*N*^ε^-lysino)-4-methyl-imidazolium
mTORC1	mammalian target of rapamycin complex 1
NAC	N-acetyl cysteine
NFATc	Nuclear factor of activated T-cells, cytoplasmic
NO	nitric oxide
NOX	NADPH oxidase
Nrf2	nuclear factor erythroid 2 related factor 2
OGTT	oral glucose tolerance test
p38 MAPK	p38 mitogen-activated protein kinase
PAI-1	plasminogen activator inhibitor 1
PARP	poly(ADP-ribose) polymerase
*Pdx1*	gene coding for pancreatic duodenal homeobox-1
PDX-1	homeodomain (HD)-containing transcription factor (syn: IPF-1 (insulin promoter factor 1)
PERK	double-stranded RNA-activated protein kinase-like endoplasmic reticulum kinase
PGC1α	transcriptional coactivator PGC1-α
PI3K	phosphatidylinositol (PI) 3-kinase
PKB/Akt	protein kinase B (serine/threonine kinase)
PKC	protein kinase C
p-JNK	phosphorylated c-Jun NH_2_—terminal kinase
p-p38	phosphorylated p38 kinase
p-ERK	phosphorylated extracellular signal-regulated kinase
PHLPP2	PH domain leucine-rich repeat protein phosphatase 2
PON1	paraoxonase 1
PPAR	peroxisome proliferation-activated receptor
RAGE	AGEs receptor
RAAS	renin-angiotensin-aldosterone system
RCS	Reactive carbonyl species
RONS	Reactive oxygen and nitrogen species
sdLDL	small dense low density lipoproteins
SD rats	Sprague Dawley rats
SHR	Spontaneously hypertensive rats
SNP	sodium nitroprusside
SSAO	semicarbazide-sensitive amine oxidase
STZ	streptozotocin
sICAM-1	soluble intercellular adhesion molecule 1
SOD-(1–3)	superoxide dismutase (1–3)
sPLA2	secreted phospholipase A 2
sRAGE	soluble RAGE produced by alternative splicing
sVCAM-1	soluble vascular cell adhesion molecule 1
TAG	triacylglycerol
TAK1	transforming growth factor-β-activated kinase 1
T1DM	type 1 diabetes
T2DM	type 2 diabetes
TGF-β	transforming growth factor β
THP	tetrahydropyrimidine
TNF-α	tumor necrosis factor α
UCP-2	uncoupling protein 2
UDPGlcNAc	uridine diphosphate N-acetylglucosamine
UPR	unfolded protein response
VCAM-1	vascular cell adhesion molecule 1
VEGF	vascular endothelial growth factor
VEGFR-2	vascular endothelial growth factor receptor 2
VSMCs	vascular smooth muscle cells
8-OHdG	8-hydroxy-2-deoxyguanosine
WKY	Wistar Kyoto rats
